# The Scientific Impact of Nations: Journal Placement and Citation Performance

**DOI:** 10.1371/journal.pone.0109195

**Published:** 2014-10-08

**Authors:** Matthew J. Smith, Cody Weinberger, Emilio M. Bruna, Stefano Allesina

**Affiliations:** 1 Department of Ecology & Evolution, University of Chicago, Chicago, Illinois, United States of America; 2 Center for Latin American Studies, University of Florida, Gainesville, Florida, United States of America; 3 Department of Wildlife Ecology and Conservation, University of Florida, Gainesville, Florida, United States of America; 4 Computation Institute, University of Chicago, Chicago, Illinois, United States of America; 5 Northern Plains Center for Human Potential, St. Paul, Minnesota, United States of America; Université de Montréal, Canada

## Abstract

International collaboration is becoming increasingly important for the advancement of science. To gain a more precise understanding of how factors such as international collaboration influence publication success, we divide publication success into two categories: journal placement and citation performance. Analyzing all papers published between 1996 and 2012 in eight disciplines, we find that those with more countries in their affiliations performed better in both categories. Furthermore, specific countries vary in their effects both individually and in combination. Finally, we look at the relationship between national output (in papers published) and input (in citations received) over the 17 years, expanding upon prior depictions by also plotting an expected proportion of citations based on Journal Placement. Discrepancies between this expectation and the realized proportion of citations illuminate trends in performance, such as the decline of the Global North in response to rapidly developing countries, especially China. Yet, most countries' show little to no discrepancy, meaning that, in most cases, citation proportion can be predicted by Journal Placement alone. This reveals an extreme asymmetry between the opinions of a few reviewers and the degree to which paper acceptance and citation rates influence career advancement.

## Introduction

In 1982, Peters & Ceci conducted a clever and mischievous study [1] in which they took recently published articles in psychology that were written by scholars at prominent institutions and changed the authors' names and affiliations to fictitious ones before resubmitting the papers to the same journals in which they were originally published. The vast majority of the resubmitted manuscripts were soundly rejected: affiliation matters. Here we investigate whether a specific feature of affiliation—the country in which an author is based [2]—influences the fate of manuscripts.

Many studies [3]–[5] have contrasted national scientific productivity (i.e., number or proportion of papers published by authors affiliated with a given country) and the impact of these publications (i.e., number or proportion of citations accrued by those papers). In general, the rationale behind these studies is that whenever a country receives a larger share of citations 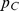 compared to the proportion of papers it publishes 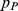, it must produce better-than-average science or science that has a greater impact. According to the same citation-centric logic, a country for which 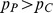 is producing sub-par science. Less well explored is the extent to which cross-national collaboration—dubbed the “fourth age of research” [6]—has transformed the creation of knowledge and the assessment of scientific productivity, but see [7].

The number of citations a country's publications receive is primarily driven by two factors: journal placement and citation performance. Journal placement, or the quality of journal into which a paper is published, influences the visibility of the papers produced: papers in certain journals, such as those with higher impact factors, often garner greater publicity and are able to attract many citations [8]. In this work, we consider the average citation rate of papers published in a given journal to be a proxy for journal quality, and use Impact Factor as a metric to quantify this rate. Specifically, we use the Impact Factor quantile of a given paper's publication outlet as its journal placement. Once a paper is published, its citation performance can be assessed as its rank (or quantile) in terms of number of citations compared to other articles published in the same journal and year [2], with such papers being of presumably similar quality in the view of a journal's editors and reviewers. Thus, by comparing papers to their peers in terms of time and publication outlet we reduce the possibility of paper quality, *per se*, confounding our results. Over-performing articles are among the top-cited for a given journal-year combination, while poorly performing ones attract a marginal number of citations compared to their peers. The proportion of citations 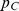 is a product of the combination of these two processes. We evaluated the effect of an author's national affiliation(s) on both journal placement and citation performance.

## Results and Discussion

**Figure 1:**
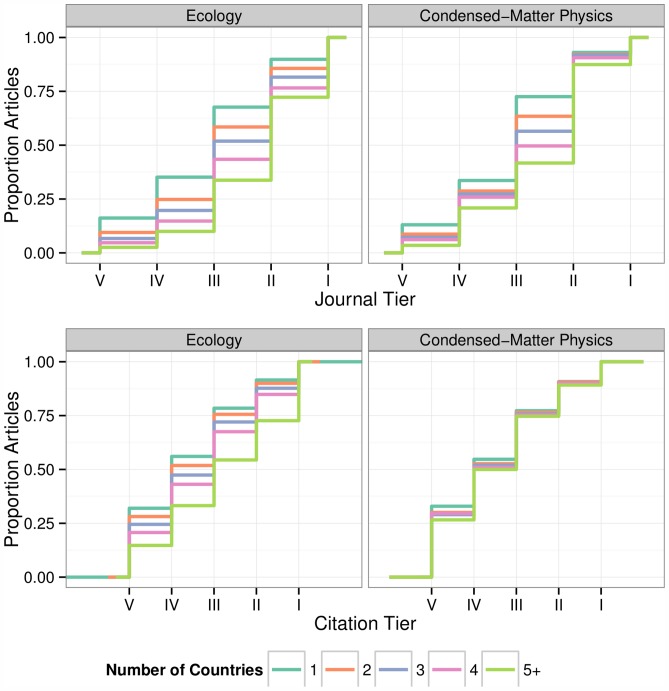
Empirical cumulative distribution function for the proportion of articles published in journals of tier V-I (top). Articles are grouped according to the number of countries included in the affiliation. (bottom) As above, but tiers are obtained binning articles in a given journal/year by citations.

We found that international collaboration has a strong, positive influence [9] on both journal placement and citation performance in most disciplines. As the number of countries represented in the author list increases, articles are more likely to be published in journals with higher impact factors (Figure 1, top) and accrue more citations than peer publications which have fewer countries represented (Figure 1, bottom, Figure S1 in File S1). Though there are some notable differences in the magnitude of the effect between these two cases. In Ecology, for instance, the effect of the number of countries is striking: more than 25% of articles with five or more countries are within the top 10% of articles by citation performance. In Condensed-Matter Physics, on the other hand, the benefit is more muted. It is important to note that the benefits of increased geographic representation are not simply a byproduct of increased author number, which is known to be correlated with increased frequency of citations [10], [11]. Rather, statistical models accounting for both number of countries and number of authors demonstrate there is a positive effect of multinational authorship (Table S3 in File S1).

This benefit is likely due to a number of mechanisms, of which we present several non-mutually exclusive ones here. First, if one considers each author as a source of publicity regarding his or her work, it follows that the more widespread the authors of a paper are, the less potential overlap in their spheres of influence and the greater potential for the research to garner attention in the popular press. For example, a French and Canadian collaboration could be featured in press releases in two countries (indeed, two continents) with a consequent increase in publication visibility. Second, multinational authorship could be indicative of research which is more generally applicable than a geographically restricted study, thereby increasing citation frequency or placement in particular journals. Consider a study comparing forests throughout the southeastern US, a similar study comparing forests in the southeastern US with forests in northern Spain would likely be addressing questions of greater generality, making findings applicable to a larger range of systems. Finally, international collaborations might be more able to obtain support, e.g., if each author were able to bring some national funds to the project or because funding agencies have funds targeted toward promoting international collaborations (e.g., NSF PIRE, FAPESP Collaborative Research Grants, and the European COST framework).

### Particular Country/Collaboration Effects

**Figure 2:**
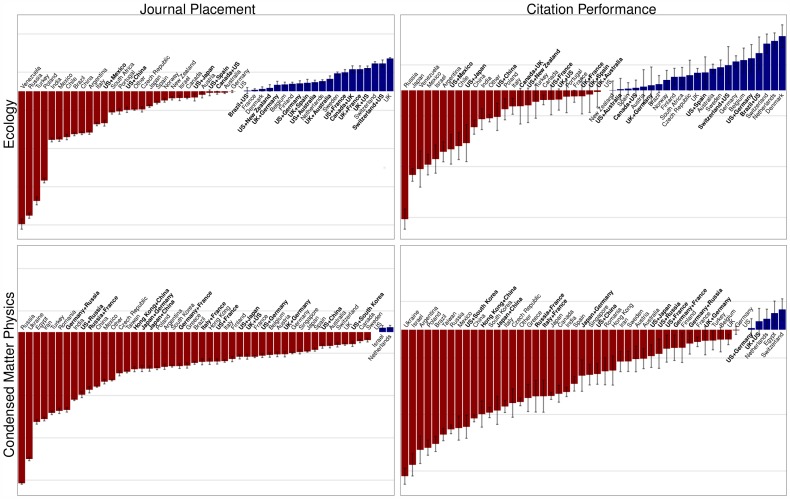
Effect of country of affiliation on journal placement and citation performance. The color and length of the bars represent the strength of the effect compared to papers originating from the US. The coefficients are obtained fitting a proportional-odds model (File S1, using a generalized linear model produces qualitatively consistent results).

Having demonstrated the overall benefits of international collaboration, we next determine if particular countries or combinations of countries deviate from the expected level of productivity and how this varies by discipline. For instance, the US is among the top-ranked countries for journal placement in Condensed-Matter Physics, Evolution, Genetics & Heredity, Geology, and Mathematics, but not in Ecology, Evolution, and Psychology. Similarly, the citation performance of the US is very strong in Physics and Genetics, but close to average in the other disciplines (Figure 2, Figure S2 in File S1). More interestingly, international collaborations can serve to improve a paper's journal placement, even beyond either individual country's performance. In Ecology, for example, US and Chinese co-authors publish articles in higher-tier journals than do Chinese authors alone (though still lower than single-country papers in the US), and Franco-American collaborations fare better than papers published by either country independently. The effect is weaker for citation performance, where we find that collaboration can be beneficial for both countries (e.g., Brazil + US), for one of the countries (Switzerland + US) or for neither (Canada + UK). Alternatively, in Condensed-Matter Physics, the effect of international collaboration seems to be more important for citations than for placement. Yet, the trend is consistent across disciplines: articles written by authors from multiple countries tend to do better than those exclusively from component countries in either placement or performance (SI).

**Figure 3:**
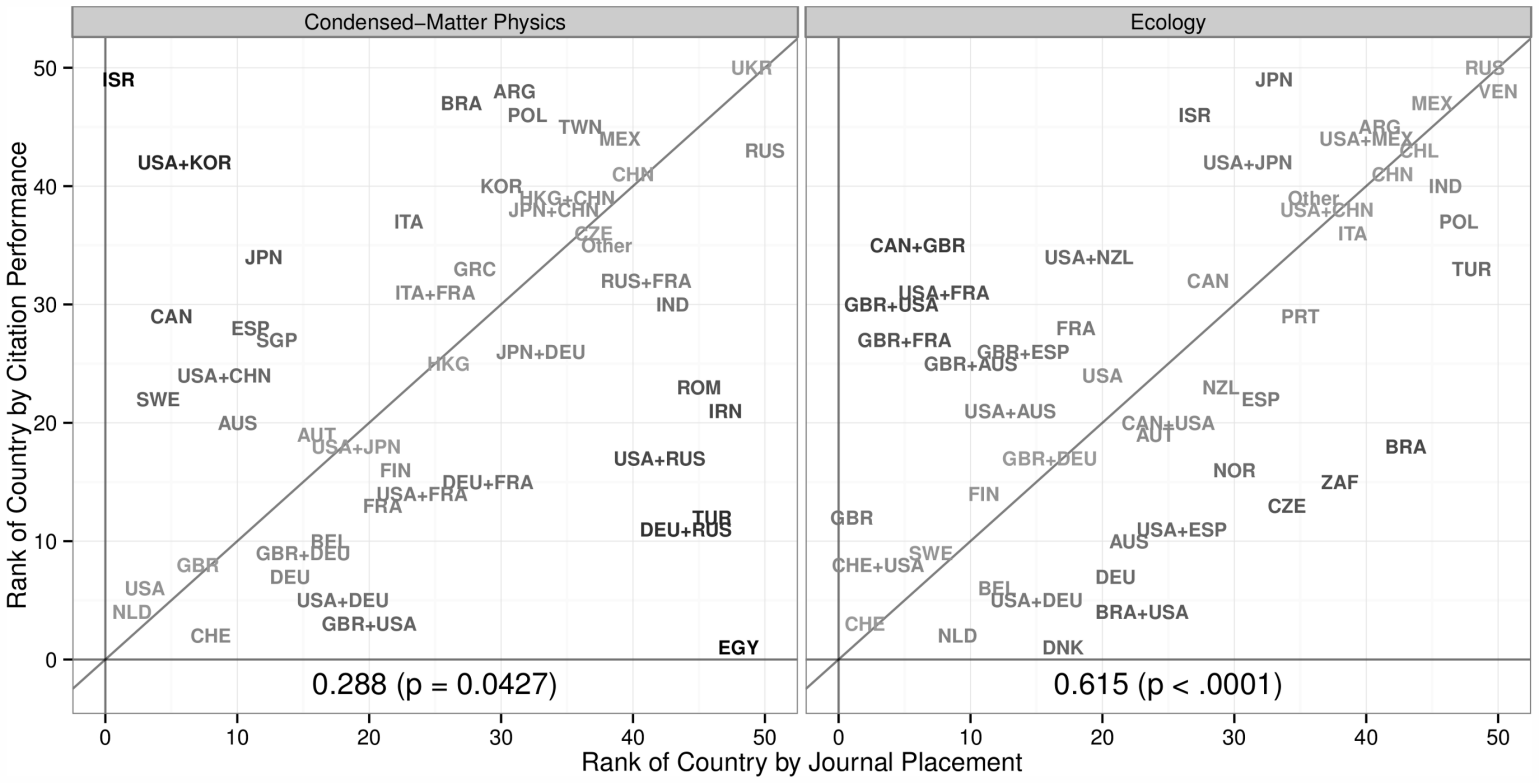
Ranking of countries in terms of journal placement vs. citation performance in a Proportional-Odds Model. Country codes (Table S4 in File S1) are shaded according to distance from the expectation of equal ranking. Under each plot is the Spearman's Rho and associated p value for the relationship.

Journal placement and citation performance of countries are correlated (Spearman Rank Correlations: 0.288, 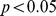 and 0.615, 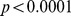 for Condensed-Matter Physics and Ecology, respectively; Figure 3, Figure S3 in File S1). Across all disciplines analyzed, Spearman Rank Correlations were significant for seven of eight using a Proportional Odds Model and five of eight using a Linear Model, with significant Spearman Rank Correlations between 0.288 and 0.615. (Figure S3 in File S1). More interesting is the case of countries that deviate markedly from this trend. For example, Brazil (in Ecology) and Egypt (in Condensed-Matter Physics) are ranked low for journal placement but high for citation performance—scientists in these countries tend to publish in lower-ranked journals, but once articles are published they tend to over-perform in terms of the number of citations they receive. Some potential explanations for this pattern include a negative bias at the peer-review stage [12] and a positive bias at the citation stage [13]. It could also be influenced by academic culture (i.e., the “biased author” effect [2]): if the academic environment in a country is such that submission to top-tier journals is discouraged, historically infrequent, or disassociated from professional advancement, then papers that could potentially be published in top journals might frequently be submitted to lower-tier ones, resulting in a mismatch between placement and performance. The same factors—bias (positive at the peer-review stage, negative at the citation stage) and academic culture (frequent submissions to top journals)—could apply to the opposite case of countries ranked highly for journals but lowly for citations (e.g., Japan and Israel in both disciplines). The nationalities of the reviewer [14] and of the author citing the work [15], as well as the authors' native language [16], likely play a role as well.

Finally, there could be macro-level drivers that influence science through funding or social acceptance, and hence affect the ability of researchers to do high-impact science [3], [4], [17]. For example, percent of GDP spent on research & development had a positive and significant effect on journal placement, while a country's GDP had a nearly universal, positive effect on both journal placement and citation performance. Interestingly, the percentage of researchers that are government employees shows a negative relationship across many fields and for both journal placement and citation performance (Table S2 in File S1).

### Publication Success Through Time

**Figure 4:**
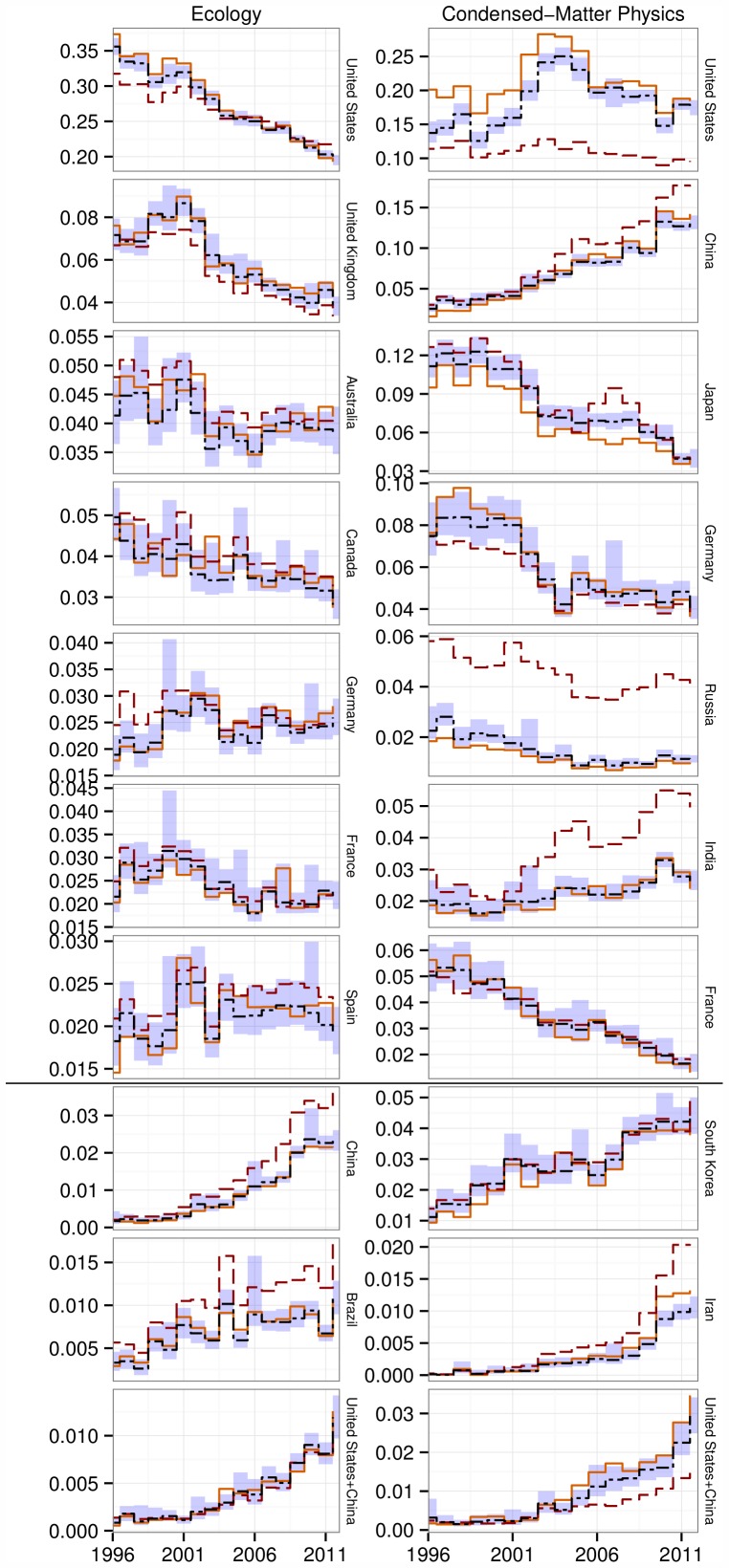
Proportion of publications and citations through time. For each year, we computed the proportion of papers published (long-dashed red line) and the proportion of citations received as of May 2013 (solid orange line). We also report the expected proportion of citations (short-dashed black line) and corresponding (95%) confidence intervals (blue shades) obtained by randomization of citation records within Journal: Year combinations. The countries displayed are, in order, the top seven by proportion of papers (above the divider) and the three fastest-rising among the remaining countries (below the divider) for Ecology (left) and Condensed Matter Physics (right).

Our analyses allow us to investigate how journal placement and citation performance contribute to the overall scientific impact of nations. To this end, we built confidence intervals for the expected number of citations received by a country in a given field and year. To build the confidence intervals, we repeatedly randomized the number of citations received by a country each year by assigning to each of a country's papers a number of citations sampled from those of “equivalent” papers, defined as those which: i) had a similar number of authors, and ii) were published in the same journal and year of the target paper (SI). In this way, we can see whether a country received more (or fewer) citations than expected when journal placement is accounted for (Figure 4, Figure S4 in File S1).

The difference between the proportion of papers (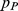, dashed red line) and the expected proportion of citations (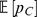, dashed black line) indicates the effect of journal placement: higher tier journals are associated with higher expected citation rates (by definition, as we assign journal rank using Impact Factors). For example, US papers in Condensed-Matter Physics tend to be published in better-than-average journals; hence, 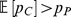, which is to say, we expect US publications in this field to receive more citations (per paper) than those same papers would if they were published in journals with lower Impact Factors. Showing the opposite trend, Russian papers tend to be published in low Impact Factor journals: 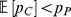. When the two lines overlap (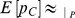, e.g., UK in Condensed-Matter Physics), the journal placement of a country tracks the overall distribution of journals. This can occur when the country is placing papers nearly uniformly across journal tiers or if high-journal-tier papers are offset by low-tier counterparts. Note that this is the case assumed by prior studies comparing 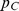 and 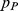 without taking journal placement into account.

The solid orange line marks the proportion of citations actually accrued by a given country in each year. From the graph, one can see that some countries (e.g., Japan and Russia) have an even lower number of citations than that predicted through the consideration of their low journal placement, i.e. their actual citation rate is less than the lower bound for the confidence interval, while others (e.g., US in Condensed-Matter Physics) largely over-perform in terms of citations—even after accounting for good journal placement. The discrepancy between this orange line and the confidence interval around the dashed black line (explained above) is due to the effect of citation performance.

Our results indicate that the publication and citation share of countries in the Global North are decreasing over time, while emerging countries such as China, India and South Korea are gaining traction. The rise of China in Condensed-Matter Physics is particularly impressive: not only has the proportion of papers and citations more than quintupled in the past 15 years, but their proportion of total citations went from being significantly fewer than expected for papers published in the late 1990s to significantly more than expected in recent years. The opposite trend is observed for the contribution to Ecology of authors based in the US: older papers are cited more than expected, while recent ones track the expectation. In yet another case, India saw a steep rise in the proportion of publications in Condensed-Matter Physics but a concurrent decrease in journal placement. Thus, the expected number of total citations for India in 2012 is similar to that in 1996, even though the proportion of papers doubled in the meantime.

### Final Remarks

For most countries 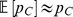 and, in general, there is more variation in journal placement than citation performance for a given field. This suggests that the total proportion of citations accrued depends more on journal placement than on the citation performance after controlling for journal and year. Given that the placement of a paper into a journal is usually based on the opinions of a limited number of individuals (ranging from a single editor for a rejection without review to some reviewers and one or more editorial board members in the case of acceptance), it is important to consider the disproportionally large effect this decision can have. Citation performance, on the other hand, can be thought of as more democratic in that it draws on the scientific community at large, with each active scientist able to contribute to a given publication's citation performance.

An important caveat to our results is that because our analyses are based on total citations received as of May 2013, these patterns could be influenced by how well papers “age”. For instance, if Ecological articles published by US authors tend to “age well”, i.e., have a a high probability of becoming “citation classics”, the performance of these papers would improve with time relative to those of other nations. An analysis tracking the growth of citations through time would further elucidate differential aging between nations. Nevertheless, our division of publication success into two categories—journal placement and citation performance—provides a novel framework with which the relative success of articles can be holistically assessed, yielding new insights into the scientific impact of individual countries and cross-national collaboration. The implications for individual scientists, funding agencies, and national governments are clear; promoting international collaboration has a number of important benefits for participating scholars [18] that ultimately translate into greater scientific visibility, quality, and impact.

## Analysis

We downloaded data from scopus.com for the 1.25 million “articles” published between 1996 and 2012 in discipline-specific journals belonging to Analytical Chemistry, Condensed-Matter Physics, Ecology, Evolutionary Biology, Genetics & Heredity, Geology, Mathematics, and Psychology (Table S1, SI Appendix A in File S1). For each article, we recorded the name of the journal in which it was published, its publication year, the number of references it cited, and the number of times the article had been cited by other papers as of May 2013. We also computed the number of authors and identified all countries found in the author affiliation list. We then used this information to assign each paper to the country or countries with which its authors were affiliated. In an important departure from previous studies of national productivity [3]–[6], [19], papers with multiple countries in their affiliation list were not assigned wholly or fractionally to each country involved. Rather, we kept a separate tally for each realized combination of countries, which allowed us to examine the performance of multi-national papers relative to single-country ones as well as detect differences among the diversity of realized collaborations. We then divided the journals in which these articles were published into five tiers based on their Impact Factor in 2011: top 10%, 10–25%, 25–50%, 50–75%, and 75–100% and assigned a citation-performance tier to each paper by binning the citations of papers published within the same journal and year into tiers (as above).

## Supporting Information

File S1 **Contains supporting information Figures S1–S4.** Figure S1. Empirical cumulative distribution functions for all fields. As Figure 1 in main text. The empirical cumulative distribution functions for journal placement (top) and citation performance (bottom) tiers have been plotted for each field. Articles are grouped according to the number of countries included in the affiliation. Figure S2. Effect of country affiliation for all fields. As figure 2 in main text. Effect of country of affiliation on journal placement and citation performance. The color and length of the bars represent the strength of the effect compared to papers originating from the US. The coefficients are obtained fitting either a proportional-odds model (top) or linear model (bottom) to either journal placement (left) or citation performance (right) for each field. Figure S3. Relationship between journal placement and citation performance in all fields. As figure 3 in main text. For each field, countries are positioned according to their ranks in journal placement and citation performance. Under each plot is the Spearman's Rho and associated p value for the relationship. In addition to the rankings for the proportional-odds model (top, shown in the main text for Ecology and Condensed-Matter Physics) the rankings for the linear model (bottom) are also plotted for each field. Figure S4. Proportion of publications and citations through time for all fields and countries. As Figure 4 in main text. Proportion of publications and citations through time. For each year, we computed the proportion of papers published (long-dashed red line) and the proportion of citations received as of May 2013 (solid orange line). We also report the expected proportion of citations (short-dashed black line) and corresponding (95%) confidence intervals (blue shades) obtained by randomization of citation records within Journal: Year combinations. All countries that have published in all eight fields analyzed are included.
(PDF)
