# Supplementary material for: The Scientific Impact of Nations: Journal Placement and Citation Performance
Source: PLoS One. 2014 Oct 8;9(10):e109195. doi: 10.1371/journal.pone.0109195 (PMC4189927; doi:10.1371/journal.pone.0109195)
Supplement: File S1 — Contains supporting information Figures S1–S4. Figure S1. Empirical cumulative distribution functions for all fields. As Figure 1 in main text. The empirical cumulative distribution functions for journal placement (top) and citation performance (bottom) tiers have been plotted for each field. Articles are grouped according to the number of countries included in the affiliation. Figure S2. Effect of country affiliation for all fields. As figure 2 in main text. Effect of country of affiliation on journal placement and citation performance. The color and length of the bars represent the strength of the effect compared to papers originating from the US. The coefficients are obtained fitting either a proportional-odds model (top) or linear model (bottom) to either journal placement (left) or citation performance (right) for each field. Figure S3. Relationship between journal placement and citation performance in all fields. As figure 3 in main text. For each field, countries are positioned according to their ranks in journal placement and citation performance. Under each plot is the Spearman's Rho and associated p value for the relationship. In addition to the rankings for the proportional-odds model (top, shown in the main text for Ecology and Condensed-Matter Physics) the rankings for the linear model (bottom) are also plotted for each field. Figure S4. Proportion of publications and citations through time for all fields and countries. As Figure 4 in main text. Proportion of publications and citations through time. For each year, we computed the proportion of papers published (long-dashed red line) and the proportion of citations received as of May 2013 (solid orange line). We also report the expected proportion of citations (short-dashed black line) and corresponding (95%) confidence intervals (blue shades) obtained by randomization of citation records within Journal: Year combinations. All countries that have published in all eight fields analyzed are included. (PDF) [file pone.0109195.s001.pdf]

Supporting Information

The Scientific Impact of Nations: Journal Placement and Citation Performance

Matthew J. Smith[1], Cody Weinberger[1], Emilio M. Bruna[2,3] & Stefano Allesina[1,4]

[1]Department of Ecology & Evolution, University of Chicago, Chicago, IL, USA
[2]Center for Latin American Studies, University of Florida, Gainesville, FL, USA
[3]Department of Wildlife Ecology and Conservation, University of Florida, Gainesville, FL, USA
[4]Computation Institute, University of Chicago, Chicago, IL, USA
[*]Corresponding author: Matthew Smith (mjsmith037@gmail.com)

Contents

1 Data Collection **3**

2 Methods/Results **4**

2.1 Number of countries and specific countries of affiliation 4

2.2 Proportions of papers and citations 6

2.3 Regressions Against National Metrics 7

3 Figures **8**

4 Tables **24**

5 Appendix A: Scopus Search Parameters **28**

1 Data Collection

For each field of interest, we extracted a list of contributing journals and their 2011 Impact Factors (IF) from Web of Science (Thomson Reuters). All journals for which an IF did not exist in 2011, which did not publish any "articles" (as defined by Scopus) in 2011, or which could not be matched to a journal within Scopus via ISSN were ignored. For each remaining journal, we downloaded from SciVerse Scopus (Elsevier) summary data of each paper published between 1996 and 2012 classified as an "article" within the Scopus database. For each paper published, we obtained a list of the Authors, the year of publication, the journal of publication, the number of citations received as of the date of download, a list of the affiliations associated with each author, and a list of the references cited in the publication. All data were downloaded between the dates of May 4th and May 28th, 2013. Due to limitations imposed by Scopus on the downloading of publication data, the number of publications retrieved from each journal for a given year were restricted to 2000. This restriction was rarely applied and likely had a minimal effect upon the conclusions drawn in this paper. Detailed instructions for data collection are available in Appendix A.

The affiliations for each publication were parsed and the identifiable countries were extracted, such that each publication was associated with a list of the unique countries found in the author affiliations. Thus, a publication resulting from a collaboration of two researchers in the United Kingdom and one in Germany would produce the country identifier [United Kingdom, Germany]. Country names were standardized according to those recognized in the CIA World Factbook and their respective iso-3 codes for representation in figures [1, 2] (Table S4). Publications for which no country names were able to be parsed were removed from the analysis; as were articles published in 2013, due to a greater potential for age to confound our analysis. Age will likely have a larger effect on the more recent papers because the error in our age estimation is a greater percentage of the paper's total age for these papers. This is a product of only having the year of publication (i.e. there could be as much as 11 months difference in age between two papers published in the same year and as little as one month between papers of consecutive years). Future studies looking at the influence of paper age at a higher resolution could better determine the magnitude of such an effect. Removing these papers also helps to reduce the potential effect of a delay before a paper is discovered and begins receiving citations. The data were further refined through the removal of publications for which no references or year of publication were listed. The number of papers collected for each field and the number remaining after refinement are summarized in Table S1.

Economic and demographic data for specific countries were obtained from the Organisation for Economic Co-operation and Development (OECD) [3]. These include: the percentage of government researchers as a percentage of the national total, the tertiary-level educational attainment

for age group 25-64 as a percentage of population (of that age group), the per capita gross domestic product (GDP), 2009 Programme for International Student Assessment scores in mathematics (proficiency levels), gross domestic expenditures on research and development as a percentage of GDP, and women researchers as a percentage of total researchers.

2 Methods/Results

Our analysis considers two complementary metrics of publication success: journal placement and citation performance. Journal placement (JP) is a measure of the quality of journal in which a given paper is published (as indicated by IF, either directly or, more powerfully, through categorization into tiers based upon quantile within all journals of a given field). Citation performance (CP) is an indication of a paper's post-publication performance given the journal and year of publication. This can be measured directly by comparing the total number of citations a paper has accrued to those of other publications of the same journal volume or by categorizing the performance by quantile within all publications of that journal-year combination. The quantiles used in both cases for this analysis were the top 10%, 10-25%, 25-50%, 50-75%, and 75-100%. This asymmetrical division was chosen to more accurately detect differences in performance given the highly skewed distributions of journal Impact Factors and paper citation rates.

2.1 Number of countries and specific countries of affiliation

That papers with multiple countries in their affiliations do better than papers with fewer is obvious from the cumulative distribution functions in Figures 1 and S1, yet these plots do not take other factors into consideration which are known to have an effect on citation rates, e.g. the number of authors [4]. To parse these effects and determine the independent influence of the number of countries found in a paper's affiliations, we fit the data to two separate statistical models: a multiple linear regression model and a proportional odds model, using the statistical software `R` and the R-package `Ordinal`, respectively [5, 6]. For each, both journal placement and citation performance were modeled. Variables taken into account in these models include the journal of publication, the age of the paper, the base-ten logarithm of the number of references cited in the paper, the number of authors, and the number of countries represented in those authors' affiliations. A two-fold approach allows the comparison of results, strengthening any conclusions drawn from these analyses. The proportional odds model was chosen in addition to the more common multiple regression to allow for greater statistical power, at the cost of more detailed information regarding specific effects of the various factors involved.

Though multiple linear regressions are ubiquitous in statistical analyses, the proportional odds

model is less well known, and thus will be briefly explained here. The proportional odds model is an ordered logit statistical model in which the data are divided into ordered response groups or tiers which are either inherent to the data or assigned prior to analysis (in our case these were the quantiles of impact factor and number of citations). These serve as the dependent variable we are seeking to explain. The distribution of the data among these groups is then assessed with respect to explanatory factors or covariates (in our case, we are most interested in the affiliated-country list) [7]. Differences in the distribution of data points (papers) for each value of the covariate (e.g. 'Canada + United Kingdom') lead to correspondingly separate coefficients (slopes) for each, with one value of the independent variable taken as a reference to determine an intercept for each tier (we used the United States). Thus, the intercept can be thought of as a baseline from which individual country affiliations deviate according to their coefficient. An important assumption of this model is that the relative effect (or 'odds ratio') of a given covariate is constant across tiers [7]. Put another way, the relative (dis)advantage given to a paper through the possession of a given country list compared to a paper from a different country is the same regardless of which particular journal or citation tier we choose for the comparison. A more complete explanation of the proportional odds model and the underlying statistics can be found in McCullagh [7] and Agresti[8].

Thus, the linear models took the form:

$\log(\text{Journal IF}) \sim$

$\text{Number of Authors} * \text{Number of Countries} + \log(\text{Number of References}) + \text{Specific Country Affiliation}$

$\log(\text{Number of Citations} + 1) \sim$

$\text{Num. Authors} * \text{Num. Countries} + \log(\text{Num. References}) + \text{Journal} + \text{Age} + \text{Spec. Country Aff.}$

And the proportional odds models:

$\text{Journal Quantile} \sim \text{Num. Authors} * \text{Num. Countries} + \log(\text{Num. References}) + \text{Spec. Country Aff.}$

$\text{Citation Quantile} \sim \text{Num. Authors} * \text{Num. Countries} + \log(\text{Num. References}) + \text{Spec. Country Aff.}$

Where the term to the left of the '$\sim$' is modeled with respect to the variables on the right. A '+' indicates a combination of elementary terms; '$*$' indicates both main effects and interactions between variables. The interaction between the number of authors and the number of countries was explicitly considered due to the link between these variables, namely: Number of Authors $\geq$ Number of Countries in most cases. The discrepancy between the two model types in the citation performance analysis is a result of the binning of citation counts into quantiles (as we have defined them), which take the journal and year into account prior to application of the proportional odds analysis.

The coefficients for the fifty most common specific country affiliations were ordered from least to greatest and plotted for each field considered (Figure S2). The United States was taken as a

reference point for each plot. Country ordering did not vary qualitatively between statistical tests (proportional odds model vs. multiple linear regression). Most countries' relative position was also conserved between response variables (JP or CP), however there are several cases in which drastic shifts are evident: Brazil in Mathematics, USA + France in Psychology, etc. See Figure S3 for a graphical representation of this shift and the discussion in the main text for potential causes of this discrepancy.

The analysis was repeated without the specific country affiliation and again with a subset of the total data in which only papers with over five authors were considered (in this latter case, the number of authors was removed from the independent variables as well). The former allowed the evaluation of whether the addition of specific-country affiliations enhanced the model (it did, both in terms of the coefficient of determination and AIC, Table S3), while the latter provided some confirmation that the effect of multiple countries is not merely a consequence of the necessarily greater number of authors (Table S3).

2.2 Proportions of papers and citations

Following the lead of previous publications [9, 10, 11], we investigated the relationship between the proportion of total papers published in a year p_P and the proportion of total citations accrued in the same year p_C, for several countries in eight separate fields. Instead of simply comparing these two numbers, however, we sought to enhance this analysis through dividing the interpretation of any discrepancy into Journal Placement (JP) and Citation Performance (CP). This interpretation is beneficial as it allows a more precise identification of the source of a discrepancy between p_P and p_C. For example, a country that consistently publishes in high-ranking journals (high JP), but receives the expected number of citations for papers in those journals (average CP) would have the same effect as a country that publishes in average journals (average JP) but outperforms other papers in those journals in terms of citations (high CP): $p_P < p_C$, despite very different sources of this inequality. To separate these two potential causes of discrepancy between p_P and p_C, we estimated the expected number of citations accrued by given country when accounting for its level of JP. This was accomplished through repeatedly randomizing the number of citations received by a country through sampling from equivalent papers. That is, for each paper published by a given country, we replaced the actual number of citations with one drawn from a list of all papers published in the same journal and year and which also had a similar number of authors (similar being defined by binning the authors into groups of 1, 2, 3, 4, 5, 6-10, 11-15, 16-20, or >20 authors). The total number of citations was then recalculated with the sampled citation counts. This process was repeated 10,000 times and a 95% confidence interval of the resulting distribution

was drawn. An analogous figure to the commonly represented line-graph comparison of p_P and p_C was then produced (Figure S4), with the p_C line now contextualized by an expected proportion of citations based on the given country's JP. It can be seen that in the majority of cases the JP completely explains the discrepancy between p_C and p_P (i.e. p_C consistently falls within the confidence interval of the expected proportion of citations). Put yet another way: we see a p_C that is not significantly different from what we would expect once we have taken the journal placement into account. Despite this majority, some cases still reveal discrepancies between the observed and expected p_C. In these cases, we can say with confidence that the number of citations is also being acted upon by CP (either positively or negatively, depending on the direction of the deviation).

2.3 Regressions Against National Metrics

Once demonstrated, the claim that certain countries and country combinations perform better than others raises the question of what is the source of inequality. Two possible interpretations include differences in key national statistics and bias. As bias is discussed within the main text, here we will elaborate upon the former hypothesis. To test the existence and effect of properties that affect publication performance, we regressed several metrics extracted from the OECD against the country-specific coefficients produced by the two statistical analyses using simple linear models of the form Coefficients $\sim$ Metric. GDP was the most consistently significant predictor of success, showing a positive relationship in all fields except Psychology for JP in both the linear model (LM) and the proportional odds model (POM). For CP, GDP showed a positive relationship in all fields for the POM, and in six of the eight fields (all but Ecology and Mathematics) for the LM. Out of the six metrics we tested, only the percentage of women researchers was never significantly regressed with the coefficients of either test. The results for the remaining five metrics are shown in Table S2.

These metrics can explain why some countries perform better than others, but are less effective at explaining why a country may have a large discrepancy between JP and CP: though there are differences in explanatory power between JP and CP for certain metrics, the sign of the effect is conserved in all cases. To explain these differences, we turn to the second hypothesis mentioned above and discussed within the main text.

3 Figures

All figures were drawn using the R-package `ggplot2` with the help of several accessory packages [12, 13, 14, 15]. Tables were generated using either the R-package `xtable` [16] or the web application at `truben.no/latex/table/`.

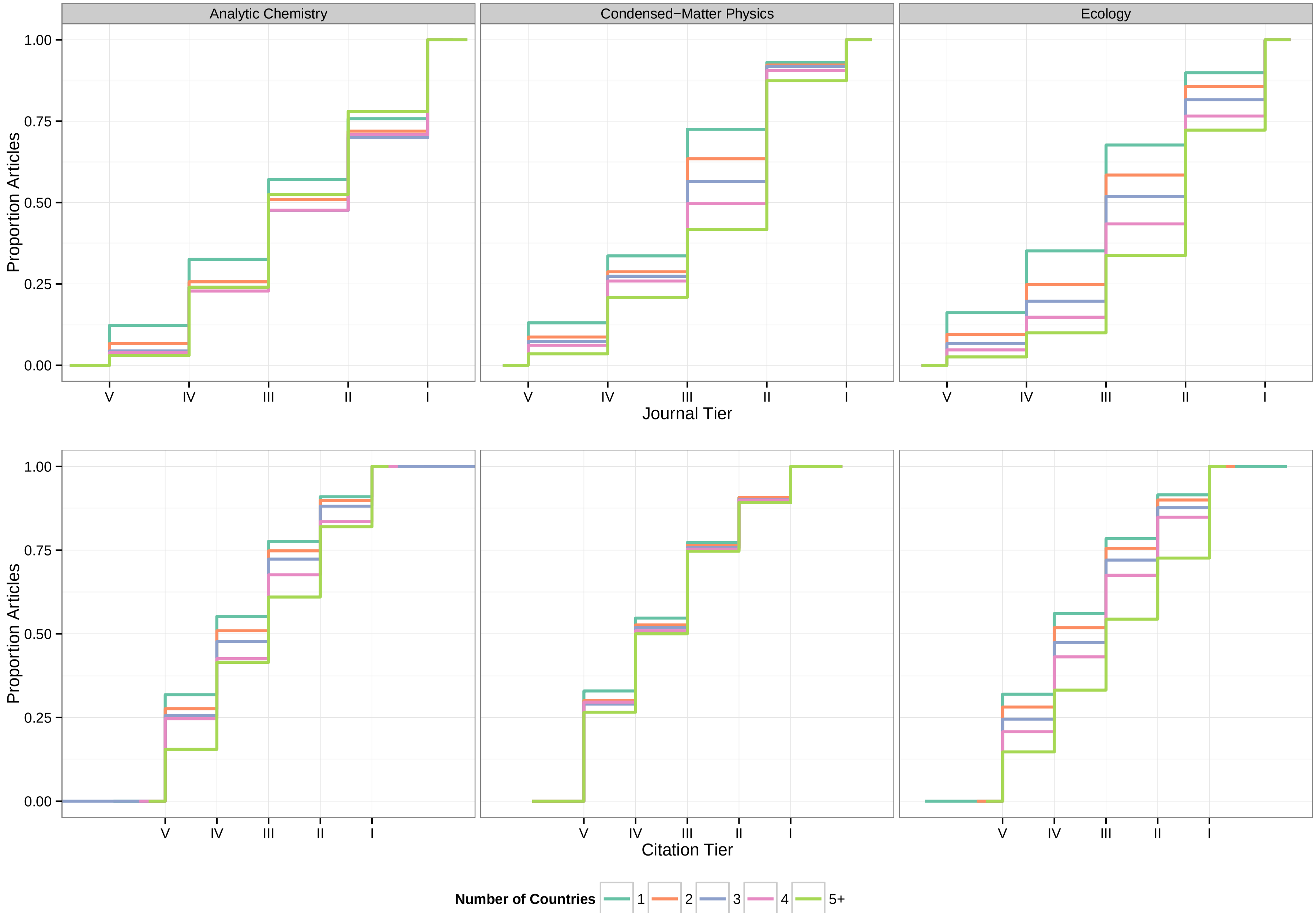

Analytic Chemistry
Condensed−Matter Physics
Ecology
Proportion Articles
1.00
0.75
0.50
0.25
0.00
V
IV
III
II
I
Journal Tier
Citation Tier
Number of Countries
1
2
3
4
5+

(a)

Figure S1

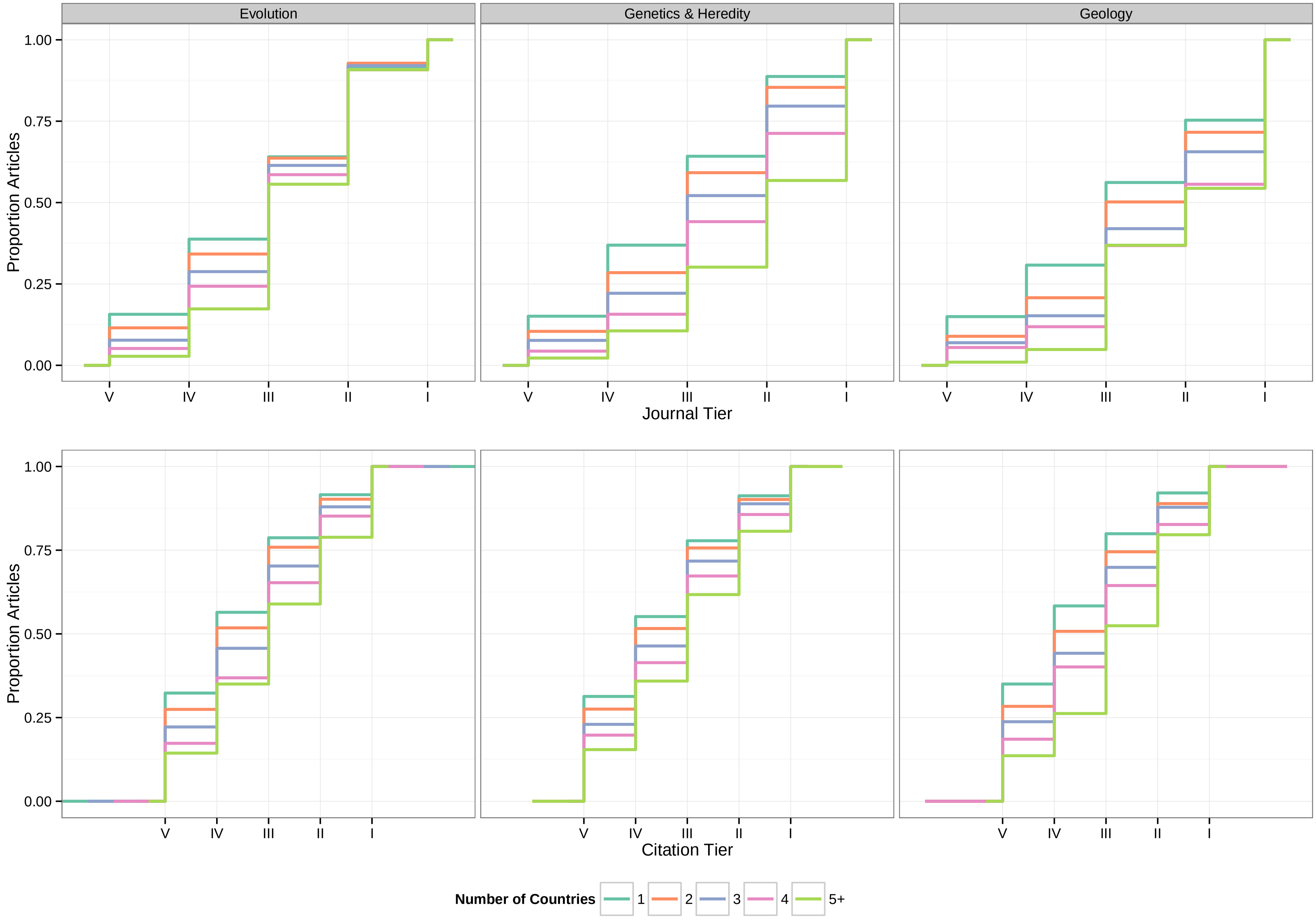
Evolution
Genetics & Heredity
Geology
Proportion Articles
1.00
0.75
0.50
0.25
0.00
V
IV
III
II
I
Journal Tier
Citation Tier
Number of Countries
1
2
3
4
5+

(b)

Figure S1

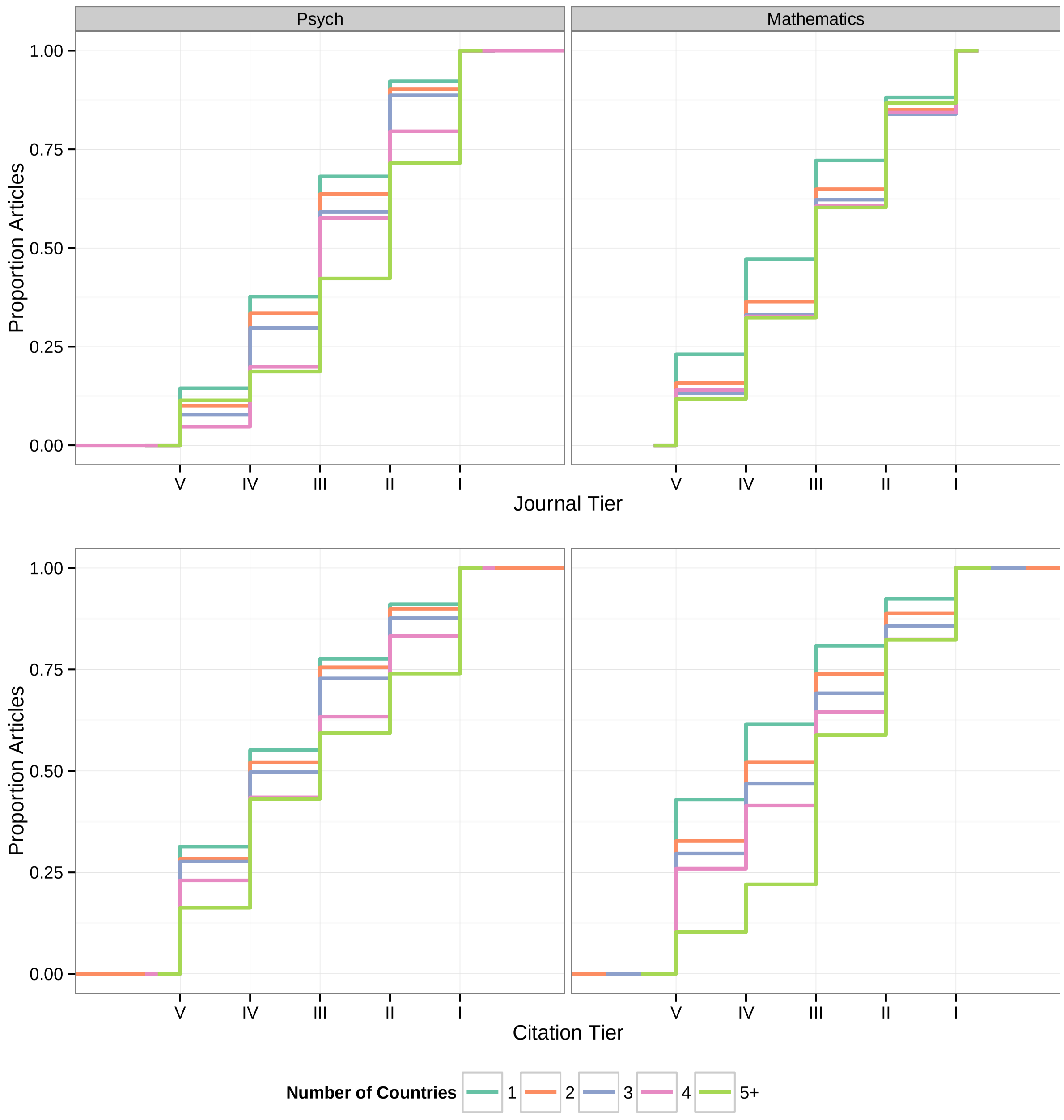

(c)

Figure S1: As Figure 1 in main text. The empirical cumulative distribution functions for journal placement (top) and citation performance (bottom) tiers have been plotted for each field. Articles are grouped according to the number of countries included in the affiliation.

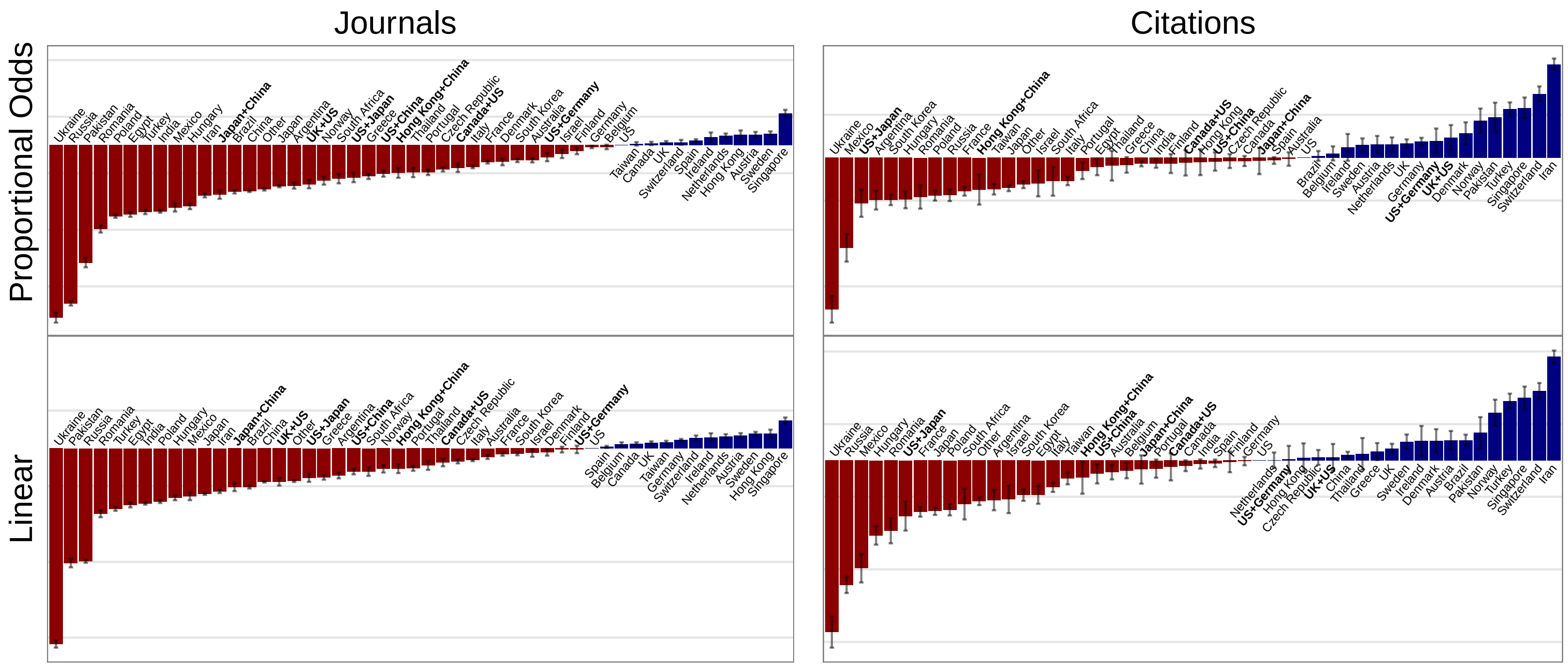

(a) Analytic Chemistry

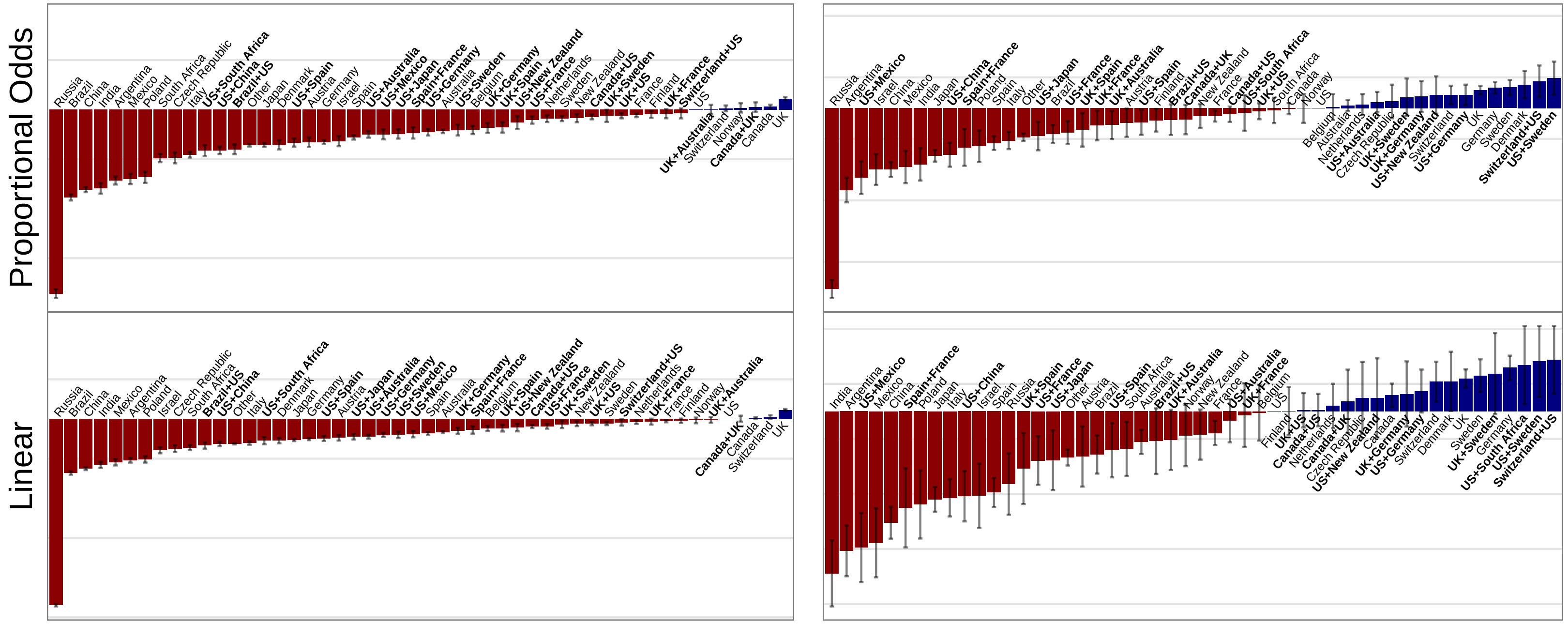

(b) Evolution

Figure S2

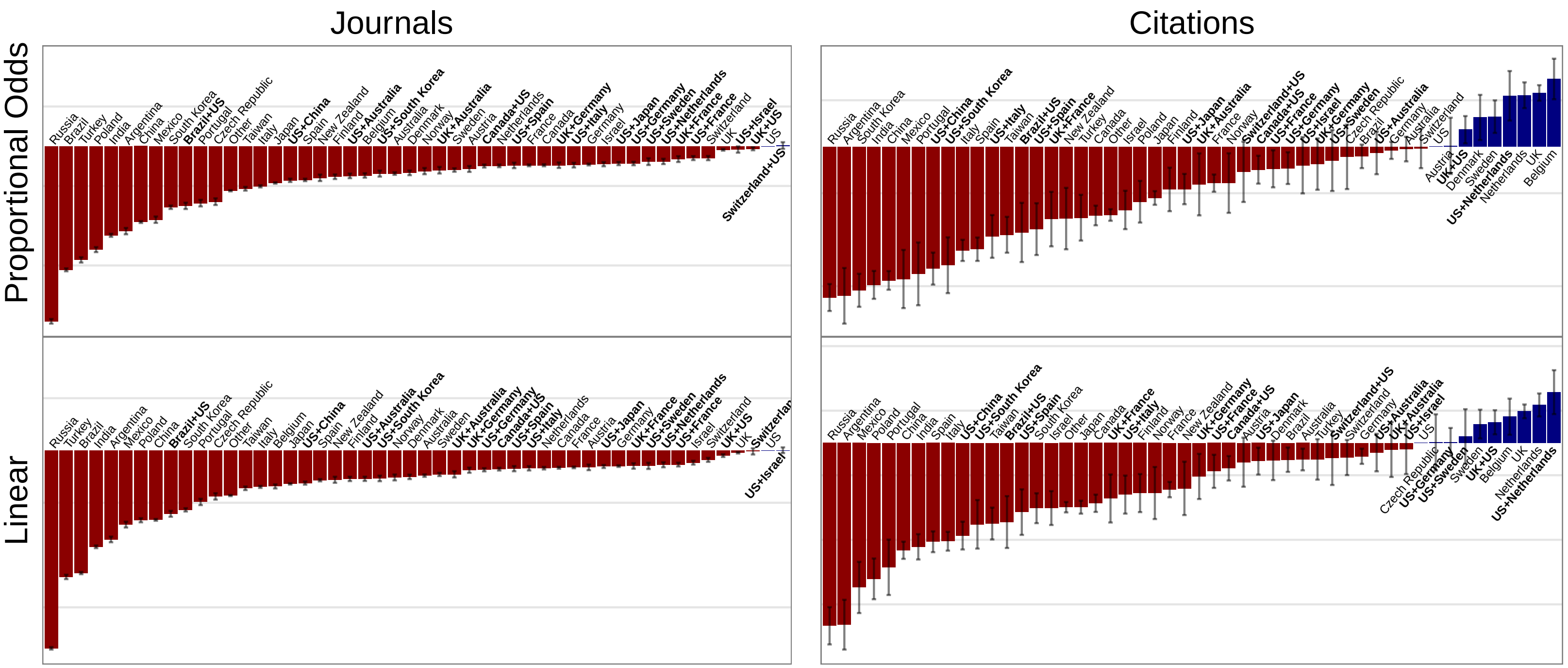

(c) Genetics & Heredity

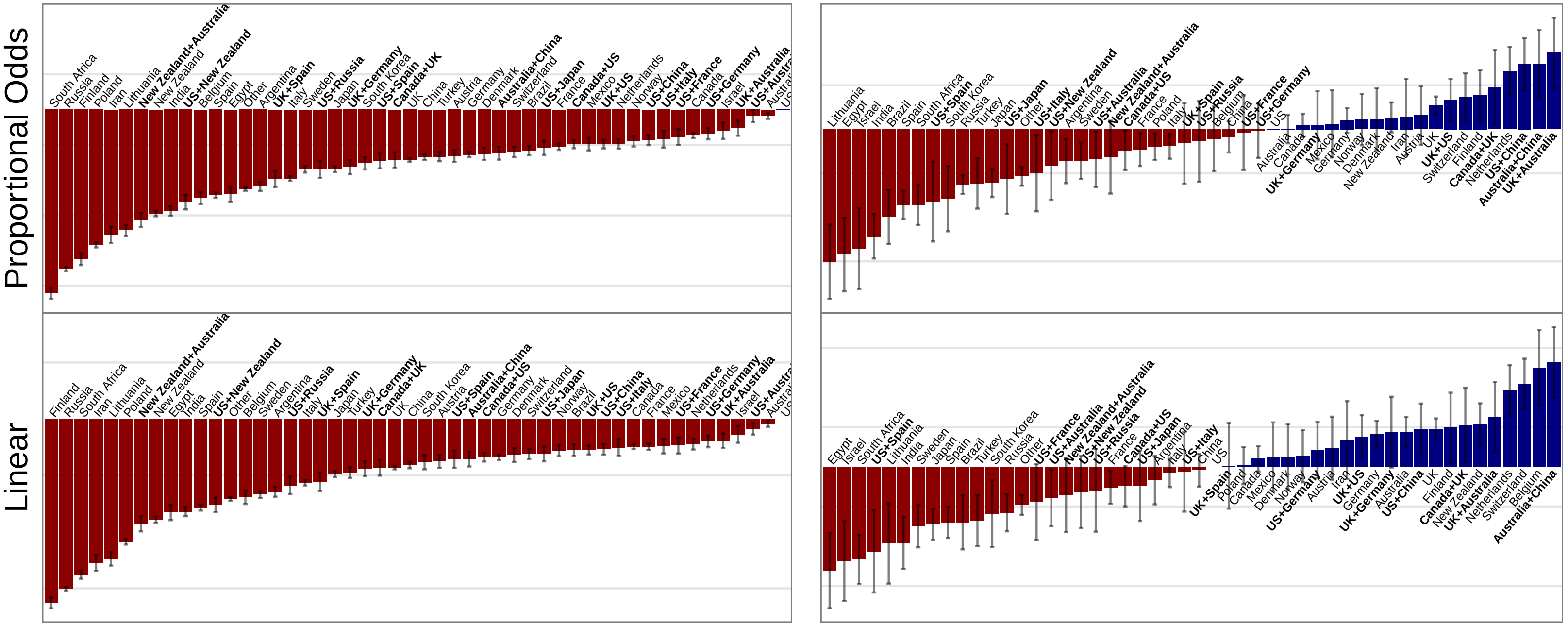

(d) Geology

Figure S2

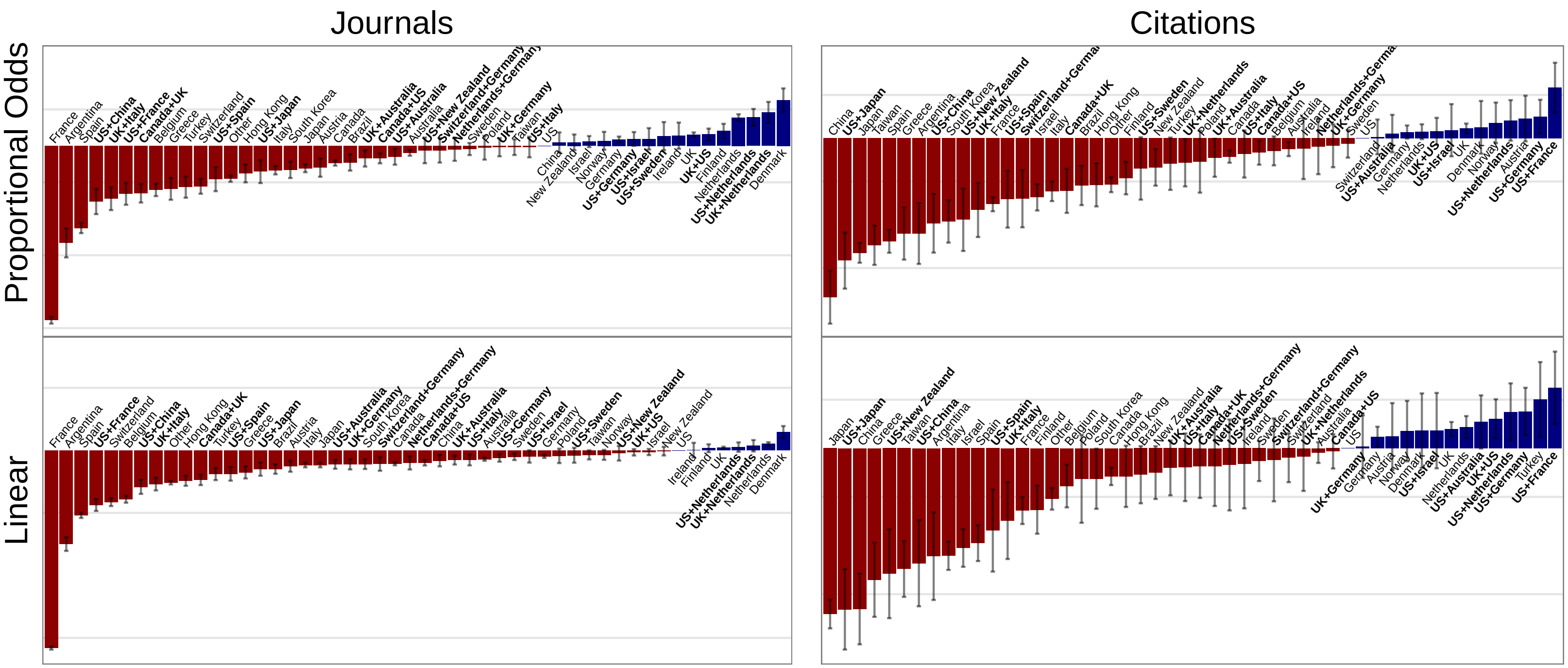

(e) Psychology

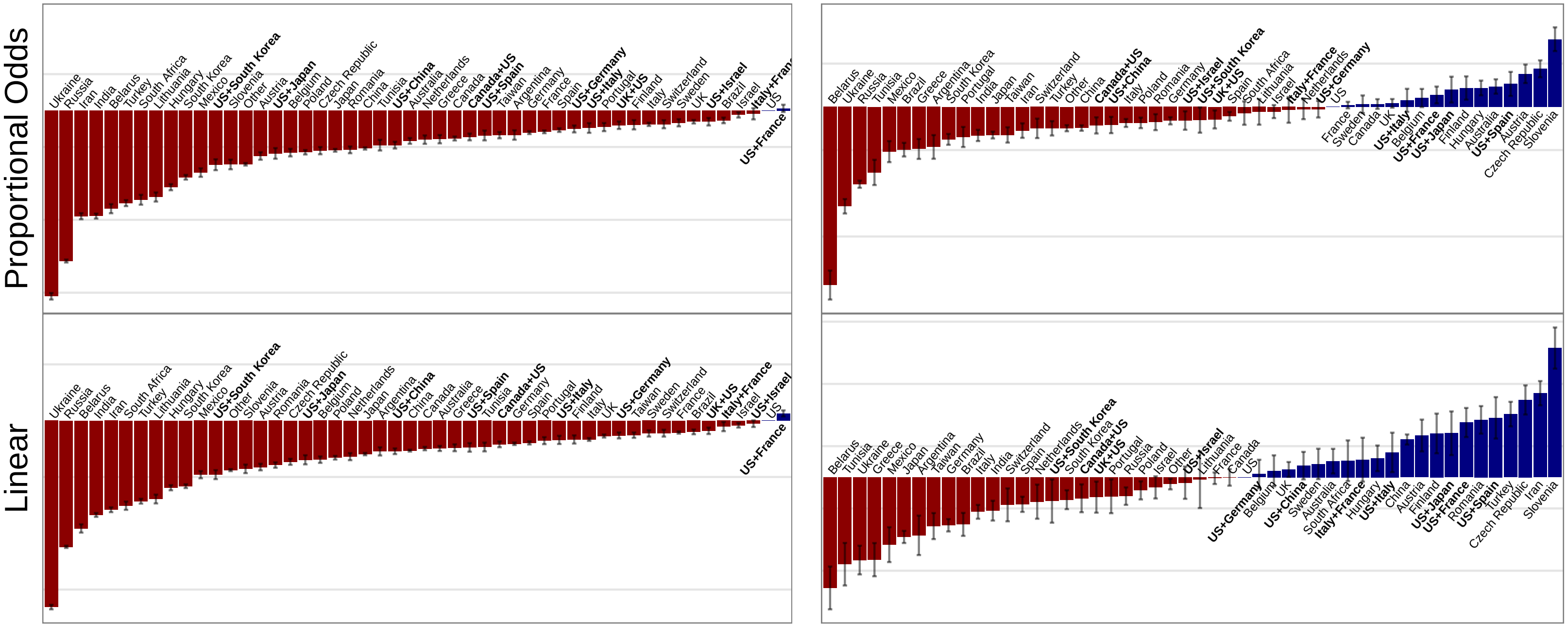

(f) Mathematics

Figure S2

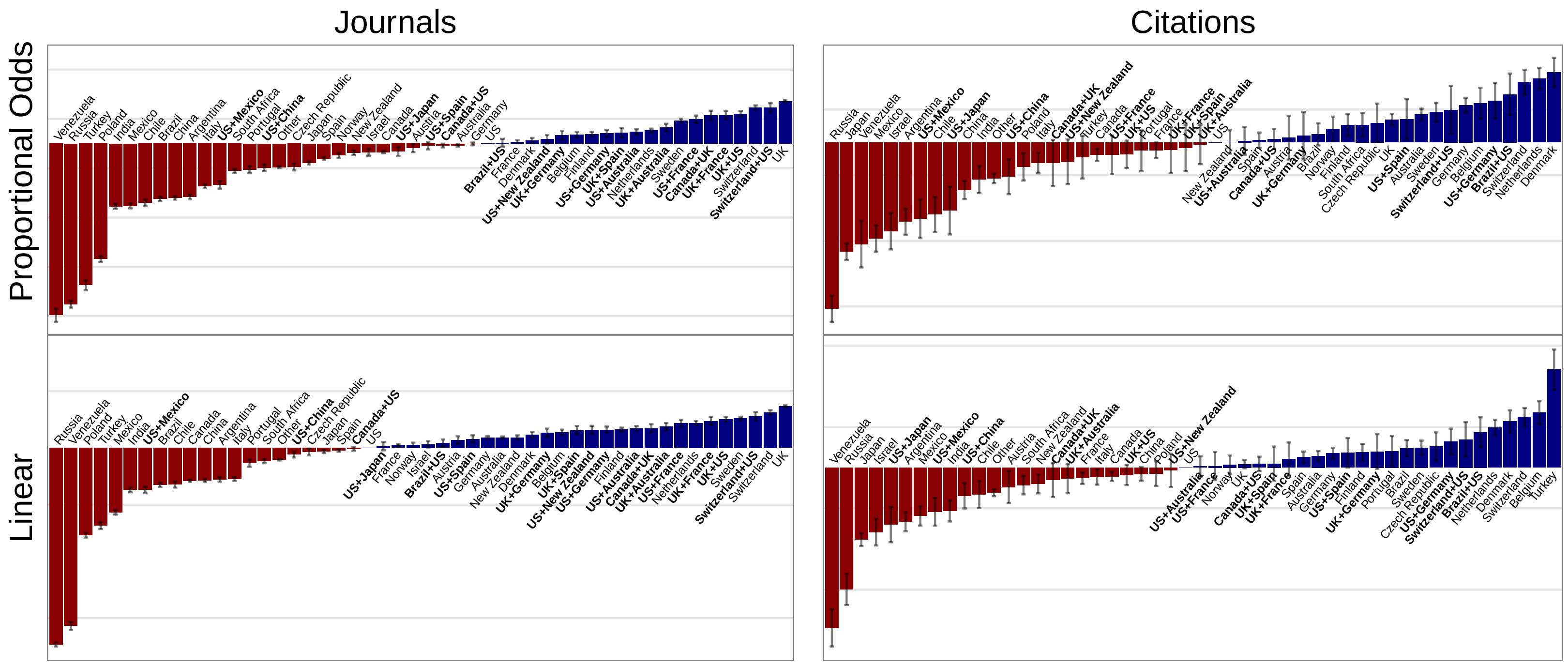

(g) Ecology

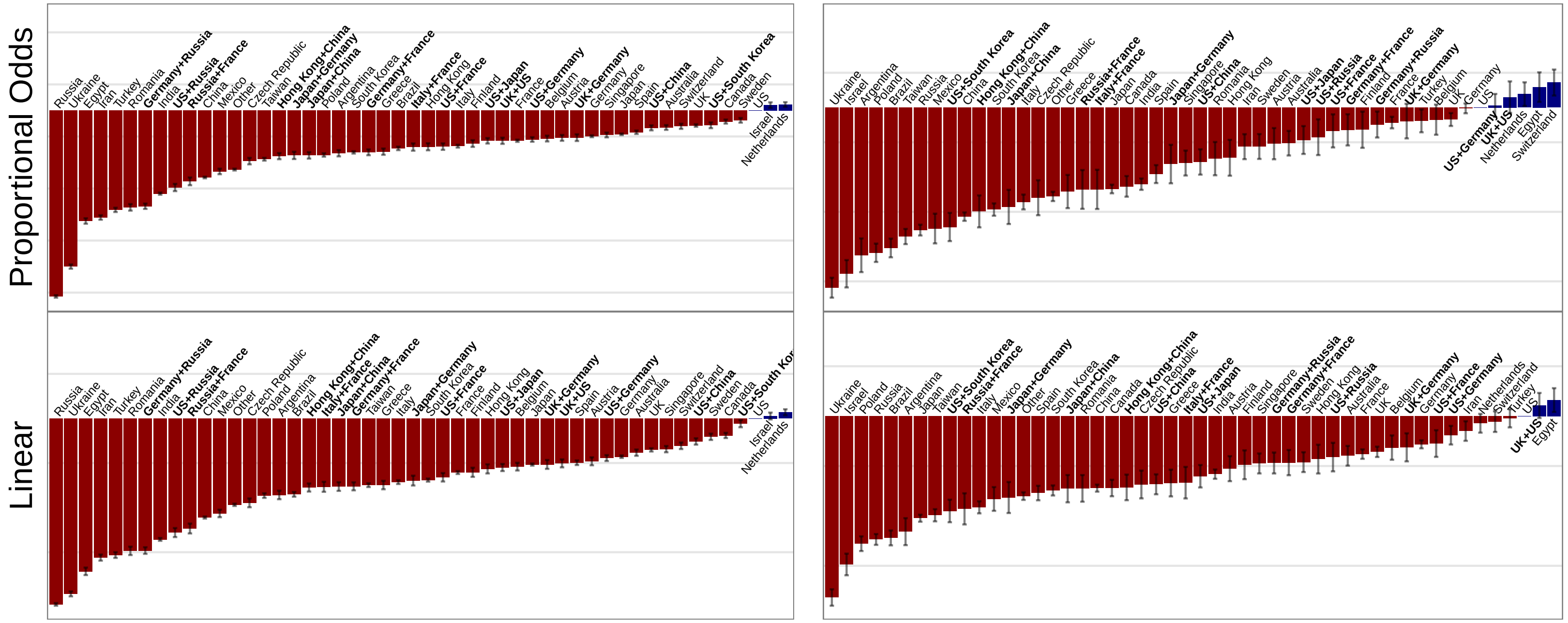

(h) Condensed-Matter Physics

Figure S2: As figure 2 in main text. Effect of country of affiliation on journal placement and citation performance. The color and length of the bars represent the strength of the effect compared to papers originating from the US. The coefficients are obtained fitting either a proportional-odds model (top) or linear model (bottom) to either journal placement (left) or citation performance (right) for each field.

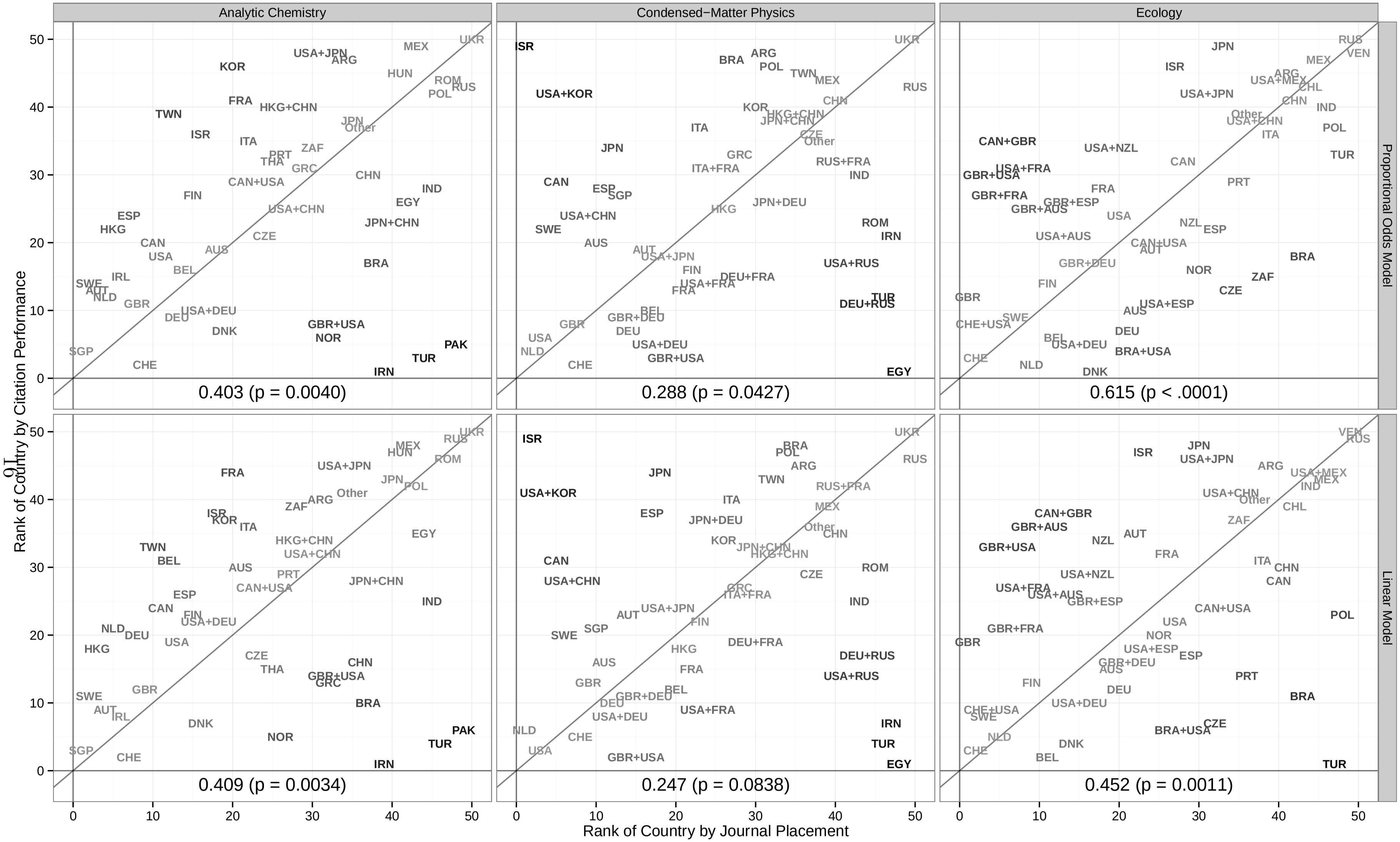
Analytic Chemistry
Condensed−Matter Physics
Ecology
Proportional Odds Model
Linear Model
Rank of Country by Citation Performance
Rank of Country by Journal Placement
0.403 (p = 0.0040)
0.288 (p = 0.0427)
0.615 (p < .0001)
0.409 (p = 0.0034)
0.247 (p = 0.0838)
0.452 (p = 0.0011)

(a)

Figure S3

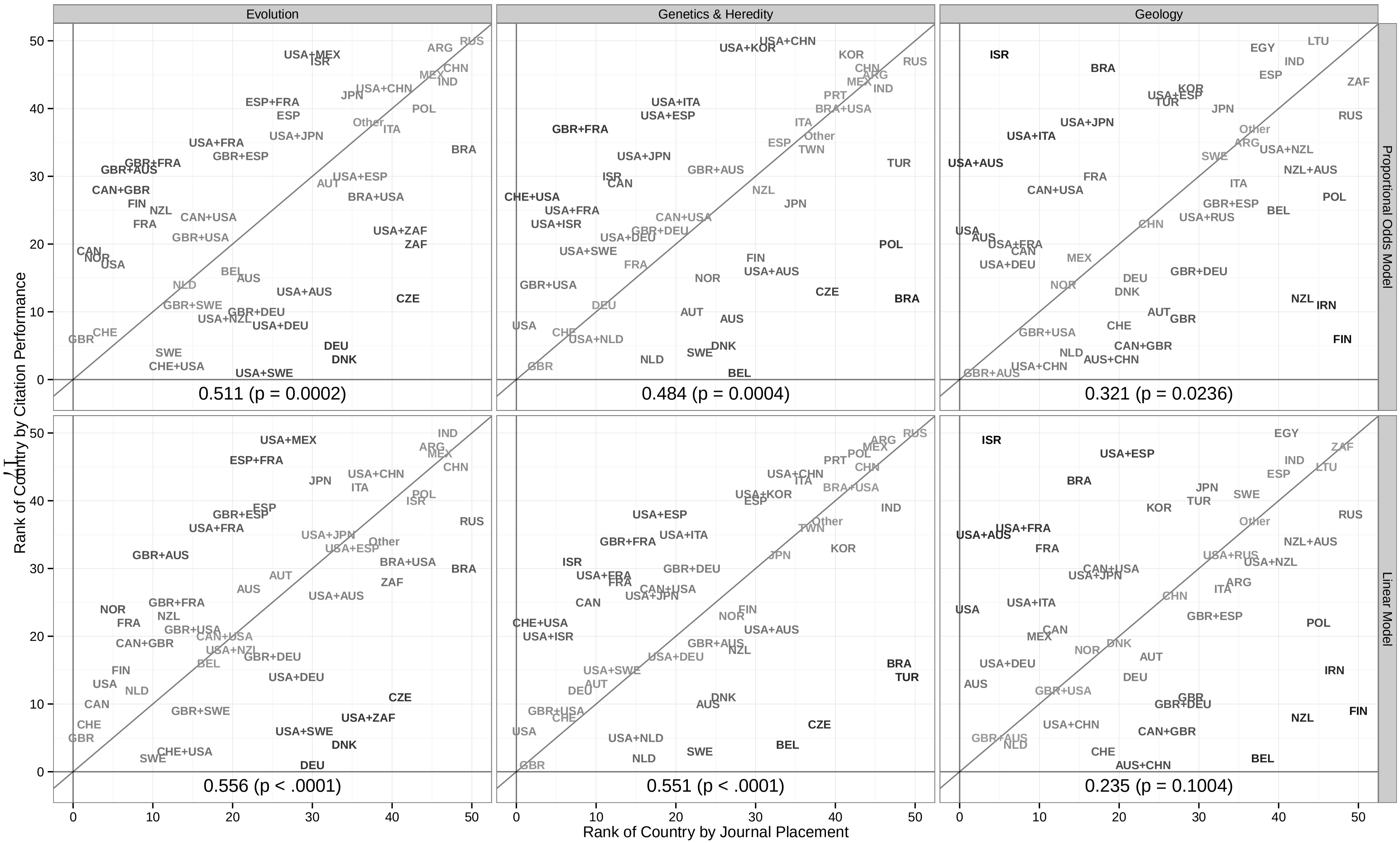
Evolution
Genetics & Heredity
Geology
Proportional Odds Model
Linear Model
0.511 (p = 0.0002)
0.484 (p = 0.0004)
0.321 (p = 0.0236)
0.556 (p < .0001)
0.551 (p < .0001)
0.235 (p = 0.1004)
Rank of Country by Citation Performance
Rank of Country by Journal Placement

(b)

Figure S3

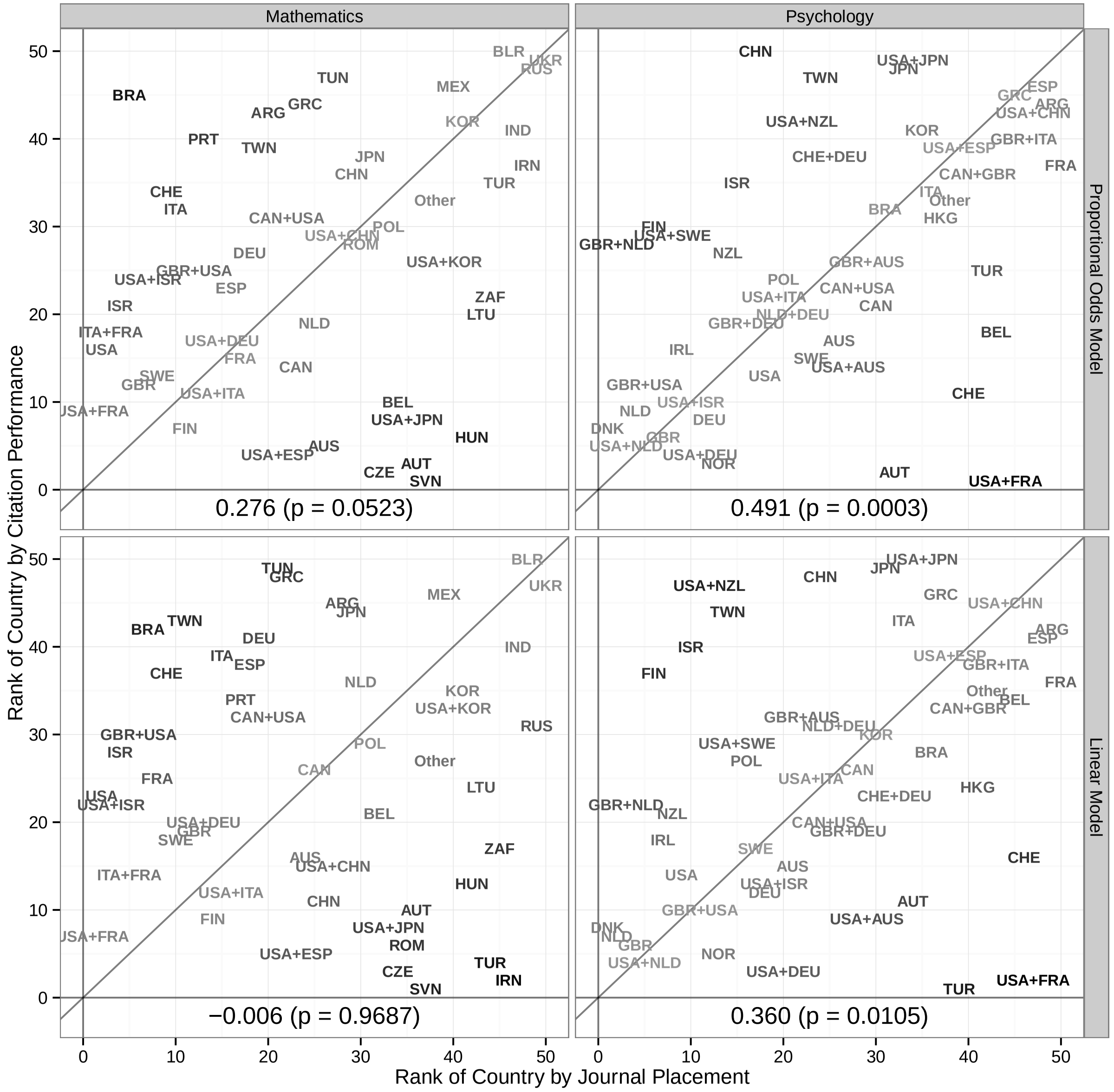

(c)

Figure S3: As figure 3 in main text. For each field, countries are positioned according to their ranks in journal placement and citation performance. Under each plot is the Spearman's Rho and associated p value for the relationship. In addition to the rankings for the proportional-odds model (top, shown in the main text for Ecology and Condensed-Matter Physics) the rankings for the linear model (bottom) are also plotted for each field.

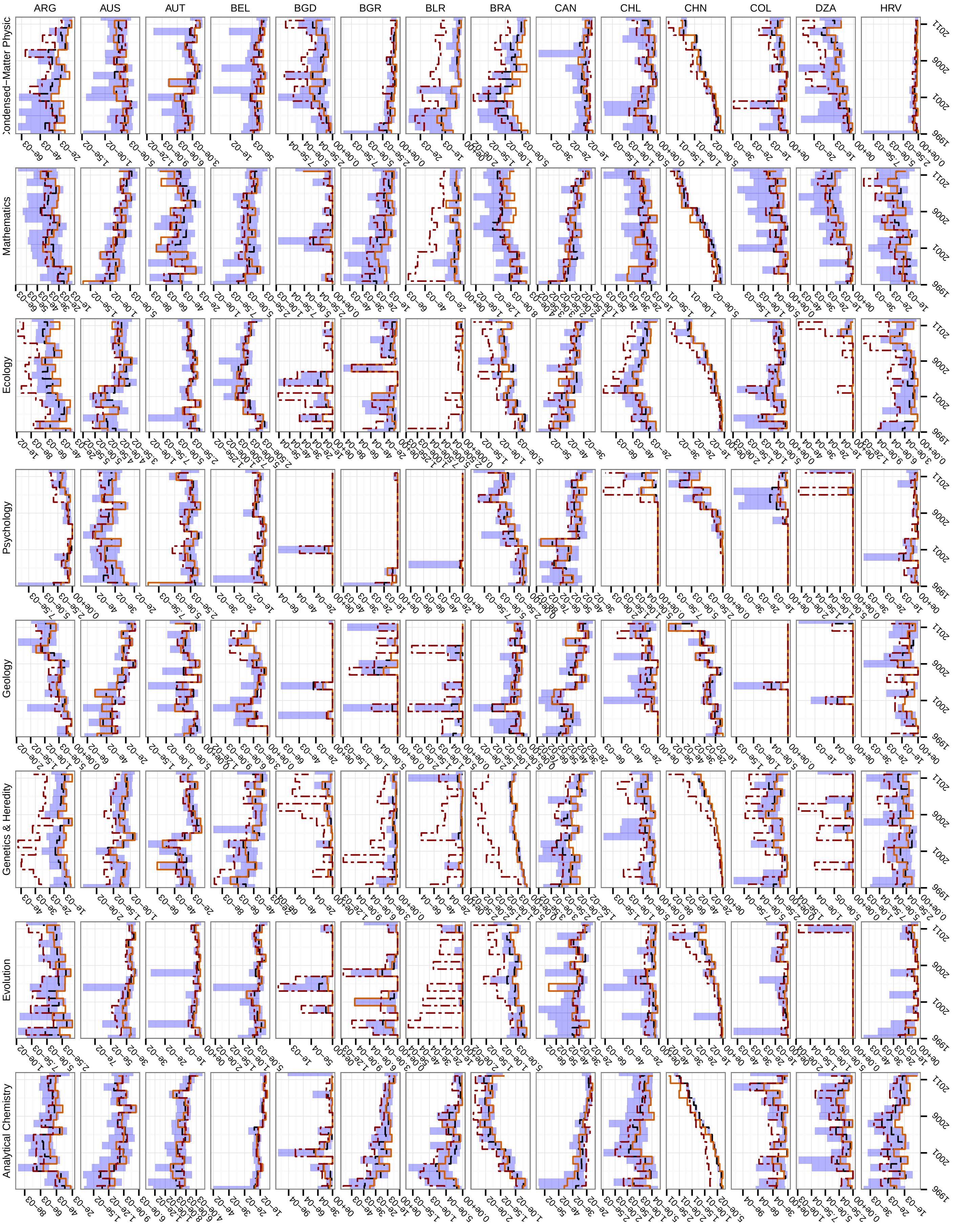
ARG
AUS
AUT
BEL
BGD
BGR
BLR
BRA
CAN
CHL
CHN
COL
DZA
HRV
Mathematics
Ecology
Psychology
Geology
Genetics & Heredity
Evolution
Analytical Chemistry

Figure S4

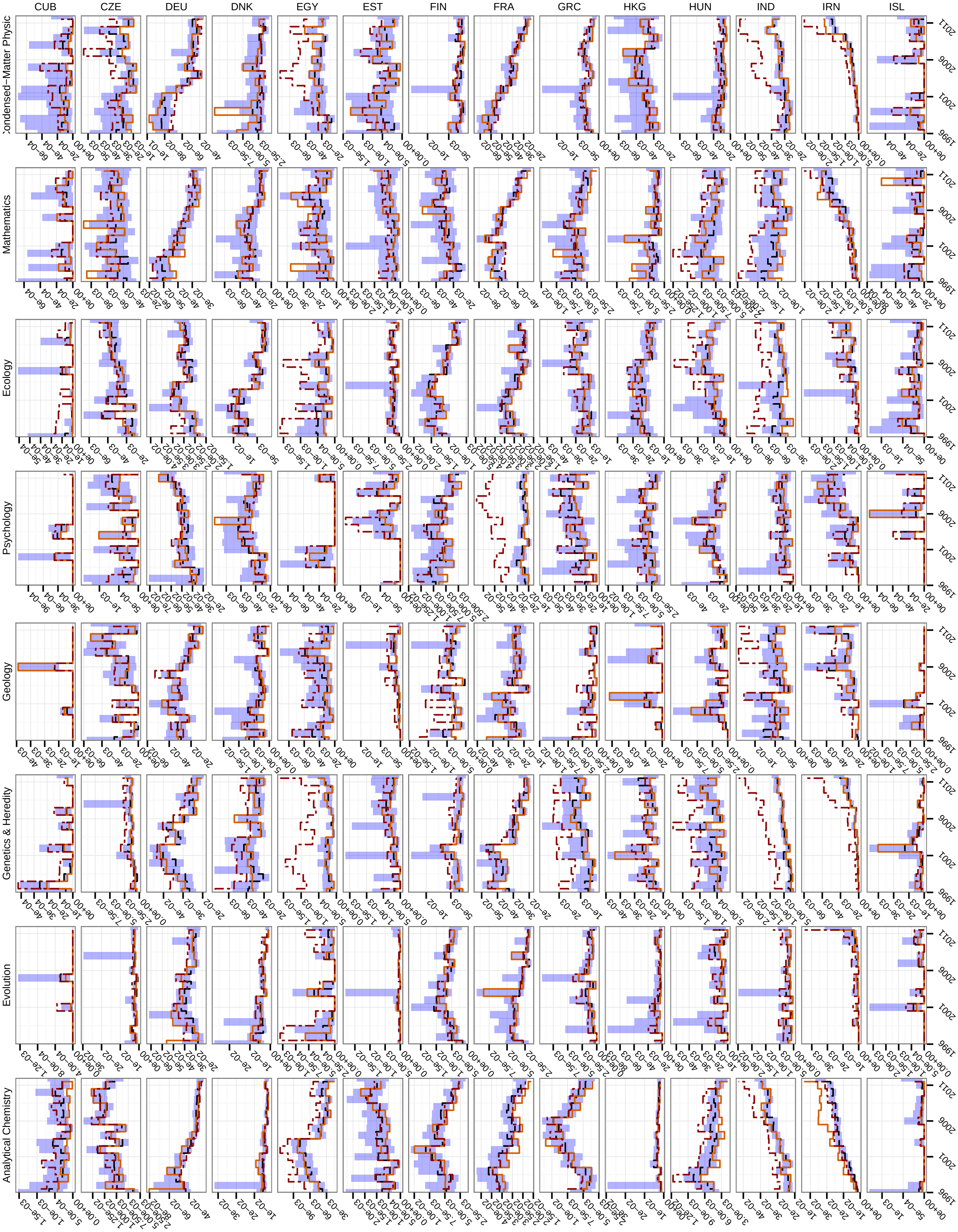
CUB
CZE
DEU
DNK
EGY
EST
FIN
FRA
GRC
HKG
HUN
IND
IRN
ISL
Condensed−Matter Physic
Mathematics
Ecology
Psychology
Geology
Genetics & Heredity
Evolution
Analytical Chemistry
1996
2001
2006
2011

Figure S4

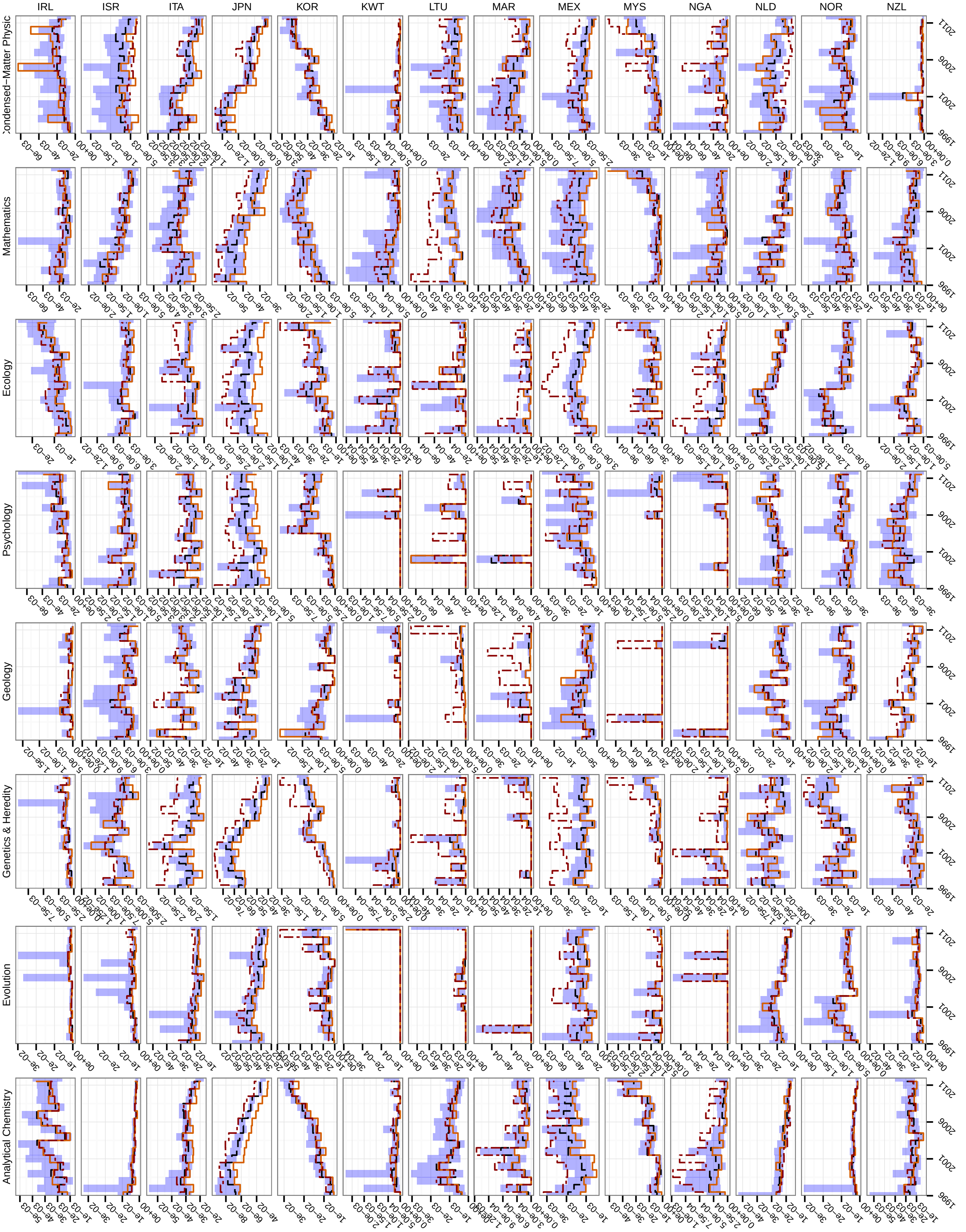
IRL
ISR
ITA
JPN
KOR
KWT
LTU
MAR
MEX
MYS
NGA
NLD
NOR
NZL
Condensed−Matter Physic
Mathematics
Ecology
Psychology
Geology
Genetics & Heredity
Evolution
Analytical Chemistry
1996
2001
2006
2011

Figure S4

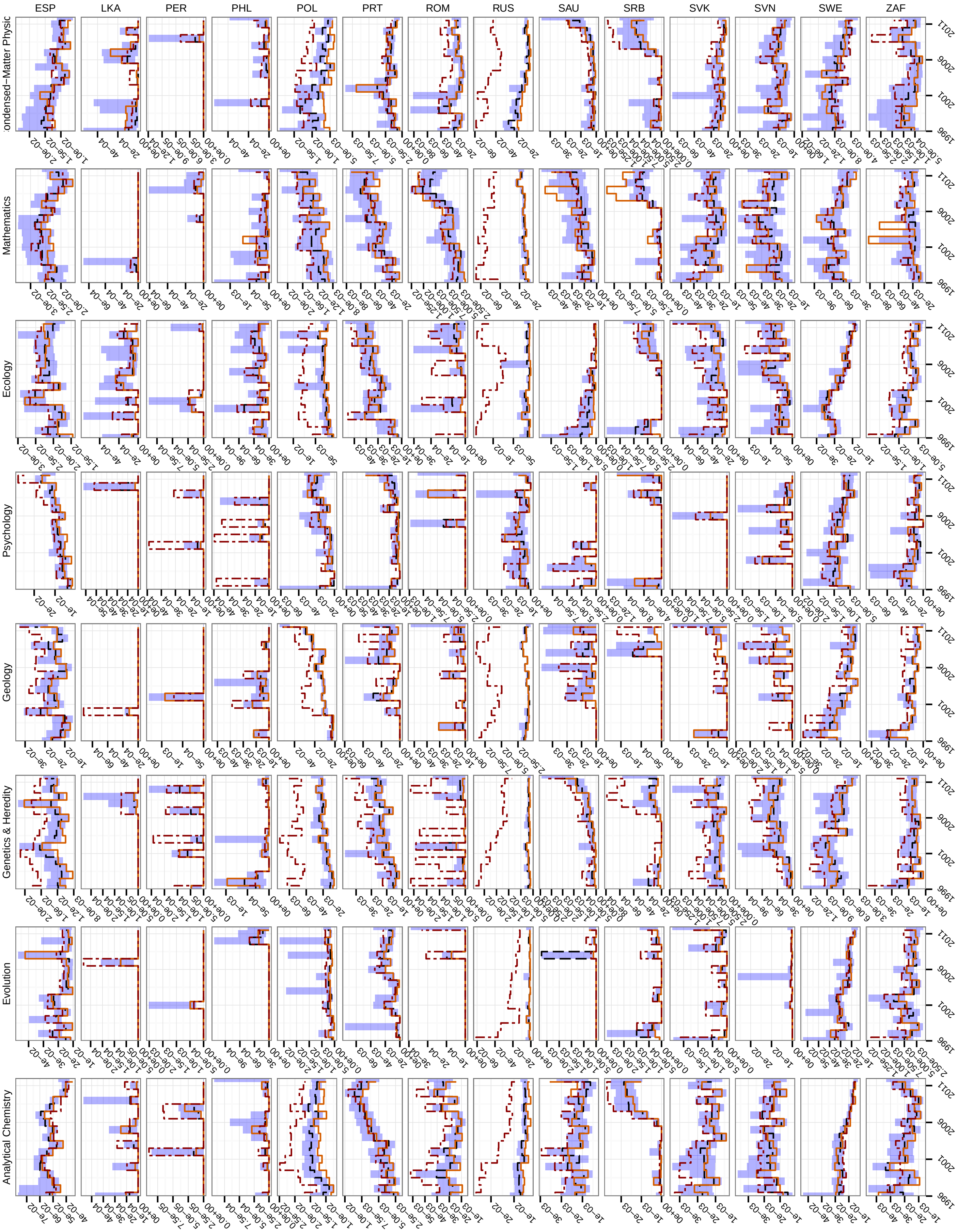
ESP
LKA
PER
PHL
POL
PRT
ROM
RUS
SAU
SRB
SVK
SVN
SWE
ZAF
Mathematics
Ecology
Psychology
Geology
Genetics & Heredity
Evolution
Analytical Chemistry

Figure S4

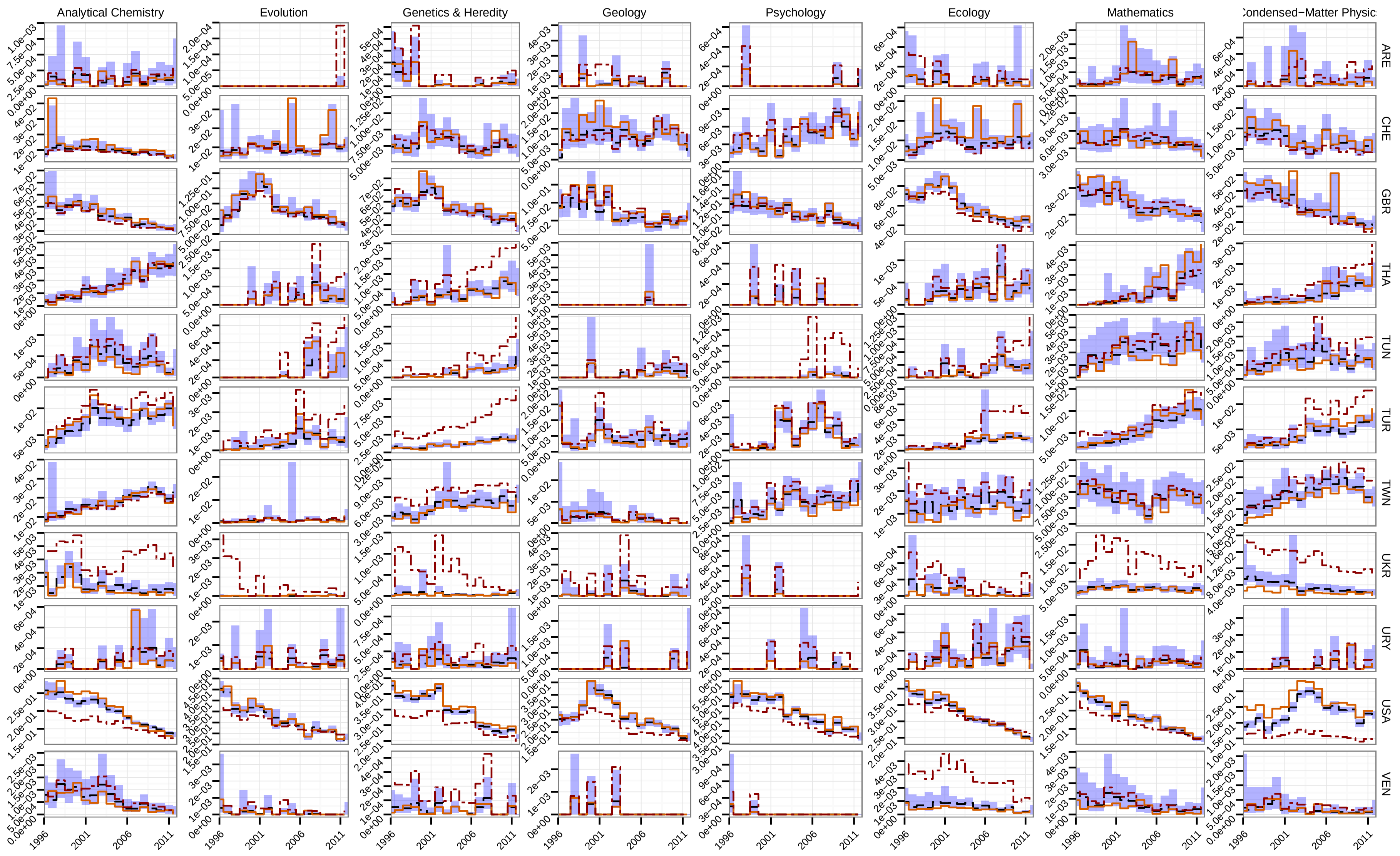

Figure S4: As Figure 4 in main text. Proportion of publications and citations through time. For each year, we computed the proportion of papers published (long-dashed red line) and the proportion of citations received as of May 2013 (solid orange line). We also report the expected proportion of citations (short-dashed black line) and corresponding (95%) confidence intervals (blue shades) obtained by randomization of citation records within Journal:Year combinations. All countries that have published in all eight fields analyzed are included.

4 Tables

Field	Number of Journals	Number of Papers	
		Raw	Refined
Condensed Matter Physics	67	290575	284208
Mathematics	255	251417	240698
Analytical Chemistry	71	224957	220092
Genetics & Heredity	146	212788	201315
Ecology	130	174258	170461
Evolution	45	61922	59253
Psychology	72	60203	58011
Geology	44	26756	25709
TOTAL	802	1247216	

Table S1: Summary of number of papers and journals per field included in this analysis.

Linear Model

Journals	Chemistry	Physics	Ecology	Evolution	Genetics	Geology	Math	Psych
GDP	+ 0.485	+ 0.652	+ 0.392	+ 0.36	+ 0.529	+ 0.214	+ 0.397	
Expend	+ 0.303	+ 0.434	+ 0.397		+ 0.265		+ 0.194	
Grad	+ 0.211	+ 0.517	+ 0.269	+ 0.382	+ 0.319		+ 0.143	
Gov	− 0.223	− 0.182	− 0.198	− 0.273				
Wom								
Pisa	+ 0.221	+ 0.186	+ 0.239	+ 0.185	+ 0.168			
Citations								
GDP	+ 0.225	+ 0.236		+ 0.426	+ 0.425	+ 0.204		+ 0.204
Expend								
Grad				+ 0.277				
Gov	− 0.222	− 0.16		− 0.35	− 0.395			
Wom								
Pisa				+ 0.376	+ 0.151			
Proportional Odds Model								
Journals								
GDP	+ 0.454	+ 0.519	+ 0.509	+ 0.518	+ 0.616	+ 0.272	+ 0.425	
Expend	+ 0.33	+ 0.39	+ 0.375		+ 0.278		+ 0.196	
Grad	+ 0.252	+ 0.481	+ 0.36	+ 0.432	+ 0.359		+ 0.134	
Gov	− 0.288		− 0.195	− 0.272	− 0.187			
Wom								
Pisa	+ 0.194	+ 0.15	+ 0.306	+ 0.225	+ 0.16			
Citations								
GDP	+ 0.251	+ 0.422	+ 0.294	+ 0.369	+ 0.462	+ 0.22	+ 0.129	+ 0.492
Expend								
Grad				+ 0.165				
Gov	− 0.201	− 0.245		− 0.288	− 0.235			
Wom								
Pisa		+ 0.164	+ 0.327	+ 0.259				

Table S2: Summary of significant ($p < 0.05$) results for linear regressions for various national statistics. Statistic codes are: GDP - *per capita* Gross Domestic Product, Expend - percent Gross Domestic Product spent on research & development, Grad - percent graduation rate, Gov - percent of researchers that are government employees, Wom - percent of researchers who are female, and Pisa - Programme for International Student Assessment (PISA) math exam scores. Sign of relationship is indicated with either '+' or '−', followed by the Spearman's Rho for the relationship.

			Baseline		With Affiliations			
Field	Statistical Model	Resp. Var.	R^2	AIC	R^2	AIC	ΔAIC	(%)
Analytical Chemistry	Linear	Journals	0.125	419639.35	0.188	403535.46	-16103.89	-3.84%
		Citations	0.418	578150.12	0.422	576521.59	-1628.53	-0.28%
	Prop. Odds	Journals		662201.26		645614.13	-16587.13	-2.50%
		Citations		658180.81		657028.35	-1152.46	-0.18%
Evolution	Linear	Journals	0.068	116252.74	0.325	97299.32	-18953.42	-16.30%
		Citations	0.535	148947.06	0.538	148698.22	-248.84	-0.17%
	Prop. Odds	Journals		177875.82		170650.01	-7225.81	-4.06%
		Citations		178005.4		177222.6	-782.8	-0.44%
Genetics & Heredity	Linear	Journals	0.108	434292.68	0.279	391363.11	-42929.57	-9.88%
		Citations	0.522	531572.65	0.524	530834.36	-738.29	-0.14%
	Prop. Odds	Journals		605948.38		576136.01	-29812.37	-4.92%
		Citations		611401.45		610482.16	-919.29	-0.15%
Geology	Linear	Journals	0.026	56260.08	0.341	46321.01	-9939.07	-17.67%
		Citations	0.368	73480.81	0.373	73361.13	-119.68	-0.16%
	Prop. Odds	Journals		79919.43		69996.78	-9922.65	-12.42%
		Citations		76344.75		76179.76	-164.99	-0.22%
Psychology	Linear	Journals	0.113	117837.73	0.317	102827.35	-15010.38	-12.74%
		Citations	0.514	154627.5	0.518	154311.16	-316.34	-0.20%
	Prop. Odds	Journals		169962.73		166157.78	-3804.95	-2.24%
		Citations		175468.85		175020.25	-448.6	-0.26%
Ecology	Linear	Journals	0.115	392632.08	0.217	371702.65	-20929.43	-5.33%
		Citations	0.544	423996.08	0.546	423428.58	-567.5	-0.13%
	Prop. Odds	Journals		506280.71		491089.68	-15191.03	-3.00%
		Citations		513980.09		513206.64	-773.45	-0.15%
Condensed-Matter Physics	Linear	Journals	0.128	640524.53	0.223	607944.71	-32579.82	-5.09%
		Citations	0.364	804716.22	0.369	802720.95	-1995.27	-0.25%
	Prop. Odds	Journals		799488.42		759781.24	-39707.18	-4.97%
		Citations		859135.93		856915.34	-2220.59	-0.26%
Mathematics	Linear	Journals	0.087	358447.37	0.166	336870.14	-21577.23	-6.02%
		Citations	0.314	602594.42	0.316	601978.75	-615.67	-0.10%
	Prop. Odds	Journals		739383.98		721226.65	-18157.33	-2.46%
		Citations		696726.86		695633.82	-1093.04	-0.16%
							Average for Journals	-7.09%
							Average for Citations	-0.20%
							Average for LM	-4.89%
							Average for POM	-2.40%

Table S3: Summary of the fit of statistical models. For each field, two models were fit (a linear model: 'Linear', 'LM', and a proportional odds model: 'Prop. Odds', 'POM'). Each model was fit to both the citation and journal tier data. Especially note the final column, which shows the percent improvement in the Akaike information criterion (AIC) when individual countries' effects are included in the model.

Country	ISO-3	English?	Country	ISO-3	English?	Country	ISO-3	English?
Algeria	DZA	No	Hong Kong	HKG	No	Portugal	PRT	No
Argentina	ARG	No	Hungary	HUN	No	Romania	ROM	No
Australia	AUS	Yes	Iceland	ISL	No	Russia	RUS	No
Austria	AUT	No	India	IND	Yes	Saudi Arabia	SAU	No
Bangladesh	BGD	No	Iran	IRN	No	Serbia	SRB	No
Belarus	BLR	No	Ireland	IRL	Yes	Slovakia	SVK	No
Belgium	BEL	No	Israel	ISR	No	Slovenia	SVN	No
Brazil	BRA	No	Italy	ITA	No	South Africa	ZAF	Yes
Bulgaria	BGR	No	Japan	JPN	No	Spain	ESP	No
Canada	CAN	Yes	Korea, South	KOR	No	Sri Lanka	LKA	No
Chile	CHL	No	Kuwait	KWT	No	Sweden	SWE	No
China	CHN	No	Lithuania	LTU	No	Switzerland	CHE	No
Colombia	COL	No	Malaysia	MYS	No	Taiwan	TWN	No
Croatia	HRV	No	Mexico	MEX	No	Thailand	THA	No
Cuba	CUB	No	Morocco	MAR	No	Tunisia	TUN	No
Czech Republic	CZE	No	Netherlands	NLD	No	Turkey	TUR	No
Denmark	DNK	No	New Zealand	NZL	Yes	Ukraine	UKR	No
Egypt	EGY	No	Nigeria	NGA	Yes	United Arab Emirates	ARE	No
Estonia	EST	No	Norway	NOR	No	United Kingdom	GBR	Yes
Finland	FIN	No	Peru	PER	No	United States	USA	Yes
France	FRA	No	Philippines	PHL	Yes	Uruguay	URY	No
Germany	DEU	No	Poland	POL	No	Venezuela	VEN	No
Greece	GRC	No						

Table S4: Lookup table for ISO-3 country codes used throughout the main text and supplementary information as well as an indication of which countries have English among their official languages.

5 Appendix A: Scopus Search Parameters

A list of field-specific journals and their Impact Factors was compiled for each of our eight fields from ISI Web of Knowledge Journal Citation Reports (Thomson Reuters). The data we analyzed can be obtained from SciVerse Scopus using an "Advanced search" of the form:

`ISSN(XXXX-XXXX) AND PUBYEAR IS YYYY AND DOCTYPE(ar)`

for each journal of interest, where `XXXX-XXXX` is replaced with a journal's ISSN and `YYYY` with a year of interest. The final term sets the document type to only return "Articles." This excludes the categories "Editorial", "Erratum", "Letter", "Note","Review", etc. and ensures that the downloaded records were original research articles, and not other types of documents. The results can be exported by first clicking on "Select all" and then "CSV export" (choosing "CSV" as the output format and "Citations, abstract and references" as the output type).

References

[1] Central Intelligence Agency. The world factbook. Accessed: May 2013.

[2] International Organization for Standardization. Country codes - iso 3166. Accessed: May 2013.

[3] Organisation for Economic Co-operation and Development. Oecd.stat.

[4] Roosa Leimu and Julia Koricheva. What determines the citation frequency of ecological papers? *Trends in Ecol Evol*, 20(1):28–32, 2005.

[5] R Development Core Team. *R: A Language and Environment for Statistical Computing*. R Foundation for Statistical Computing, Vienna, Austria, 2011. ISBN 3-900051-07-0.

[6] R. H. B. Christensen. ordinal—regression models for ordinal data, 2-09. R package version 2012.09-11 http://www.cran.r-project.org/package=ordinal/.

[7] Peter Mccullagh. Regression Models for Ordinal Data. *J R Stat Soc Series B Stat Methodol*, 42(2):109–142, 1980.

[8] Alan Agresti. Tutorial on modeling ordered categorical response data. *Psychol Bull*, 105(2):290–301, March 1989.

[9] R. M. May. The scientific wealth of nations. *Science*, 275(5301):793–796, 1997.

[10] D. A. King. The scientific impact of nations. *Nature*, 430(6997):311–316, 2004.

[11] L. Bornmann and L. Leydesdorff. Macro-indicators of citation impacts of six prolific countries: Incites data and the statistical significance of trends. *PLoS One*, 8(2), 2013.

[12] Hadley Wickham. *ggplot2: elegant graphics for data analysis*. Springer New York, 2009.

[13] Wickham and Hadley. Reshaping data with the reshape package. *Journal of Statistical Software*, 21(12), 2007.

[14] Hadley Wickham. *stringr: Make it easier to work with strings.*, 2012. R package version 0.6.2.

[15] Baptiste Auguie. *gridExtra: functions in Grid graphics*, 2012. R package version 0.9.1.

[16] David B. Dahl. *xtable: Export tables to LaTeX or HTML*, 2013. R package version 1.7-1.
